# The role of lung volume recruitment therapy in neuromuscular disease: a narrative review

**DOI:** 10.3389/fresc.2023.1164628

**Published:** 2023-07-26

**Authors:** Nicole L. Sheers, Rachel O’Sullivan, Mark E. Howard, David J. Berlowitz

**Affiliations:** ^1^ Department of Respiratory and Sleep Medicine, Austin Health, Heidelberg, VIC, Australia; ^2^ Institute for Breathing and Sleep, Heidelberg, VIC, Australia; ^3^ Department of Physiotherapy, Faculty of Medicine, Dentistry and Health Sciences, The University of Melbourne, Parkville, VIC, Australia; ^4^ Department of Physiotherapy, Christchurch Hospital, Canterbury, New Zealand; ^5^ Department of Medicine, Faculty of Medicine, Dentistry and Health Sciences, The University of Melbourne, Parkville, VIC, Australia; ^6^ Turner Institute of Brain and Mental Health, Monash University, Clayton, VIC, Australia; ^7^ Department of Physiotherapy, Austin Health, Heidelberg, VIC, Australia

**Keywords:** neuromuscular disease, amyotrophic lateral sclerosis, muscular dystrophy, lung volume recruitment, lung inflation, insufflation, breath stacking

## Abstract

Respiratory muscle weakness results in substantial discomfort, disability, and ultimately death in many neuromuscular diseases. Respiratory system impairment manifests as shallow breathing, poor cough and associated difficulty clearing mucus, respiratory tract infections, hypoventilation, sleep-disordered breathing, and chronic ventilatory failure. Ventilatory support (i.e., non-invasive ventilation) is an established and key treatment for the latter. As survival outcomes improve for people living with many neuromuscular diseases, there is a shift towards more proactive and preventative chronic disease multidisciplinary care models that aim to manage symptoms, improve morbidity, and reduce mortality. Clinical care guidelines typically recommend therapies to improve cough effectiveness and mobilise mucus, with the aim of averting acute respiratory compromise or respiratory tract infections. Moreover, preventing recurrent infective episodes may prevent secondary parenchymal pathology and further lung function decline. Regular use of techniques that augment lung volume has similarly been recommended (volume recruitment). It has been speculated that enhancing lung inflation in people with respiratory muscle weakness when well may improve respiratory system “flexibility”, mitigate restrictive chest wall disease, and slow lung volume decline. Unfortunately, clinical care guidelines are based largely on clinical rationale and consensus opinion rather than level A evidence. This narrative review outlines the physiological changes that occur in people with neuromuscular disease and how these changes impact on breathing, cough, and respiratory tract infections. The biological rationale for lung volume recruitment is provided, and the clinical trials that examine the immediate, short-term, and longer-term outcomes of lung volume recruitment in paediatric and adult neuromuscular diseases are presented and the results synthesised.

## Introduction

Respiratory muscle weakness, shallow or laboured breathing, weak cough, inability to clear oral secretions or mucus, chest infections, and/or sleep-disordered breathing are common consequences and complications that many people living with a neuromuscular disease (NMD) experience (1–5). Respiratory impairment and the associated clinical manifestations cause substantial discomfort and disability. In paediatric onset NMDs, respiratory muscle weakness can exacerbate and prolong the normal respiratory inefficiencies of infancy and may impair pulmonary and chest wall development (1). Ultimately, respiratory failure is the leading cause of death for people with conditions such as amyotrophic lateral sclerosis/motor neurone disease (ALS/MND), spinal muscular atrophy (SMA), and other muscular dystrophies (2, 3, 6).

The cornerstone of management for respiratory failure in NMD is home mechanical ventilation, most commonly non-invasive ventilation (NIV). This effective therapy can slow the progression of respiratory muscle weakness and lung volume decline, improve symptoms of breathlessness and nocturnal hypoventilation, and prolong survival in ALS/MND (4, 5, 7–10). In children, NIV can also reduce the inefficient paradoxical abdominal breathing pattern frequently present (11), and in doing so, may positively impact pulmonary growth and chest wall development (12–14). Treating hypoventilation with NIV is a critical component of respiratory care for people with NMD, but comprehensive respiratory care is broader than just NIV.

**Figure 1:**
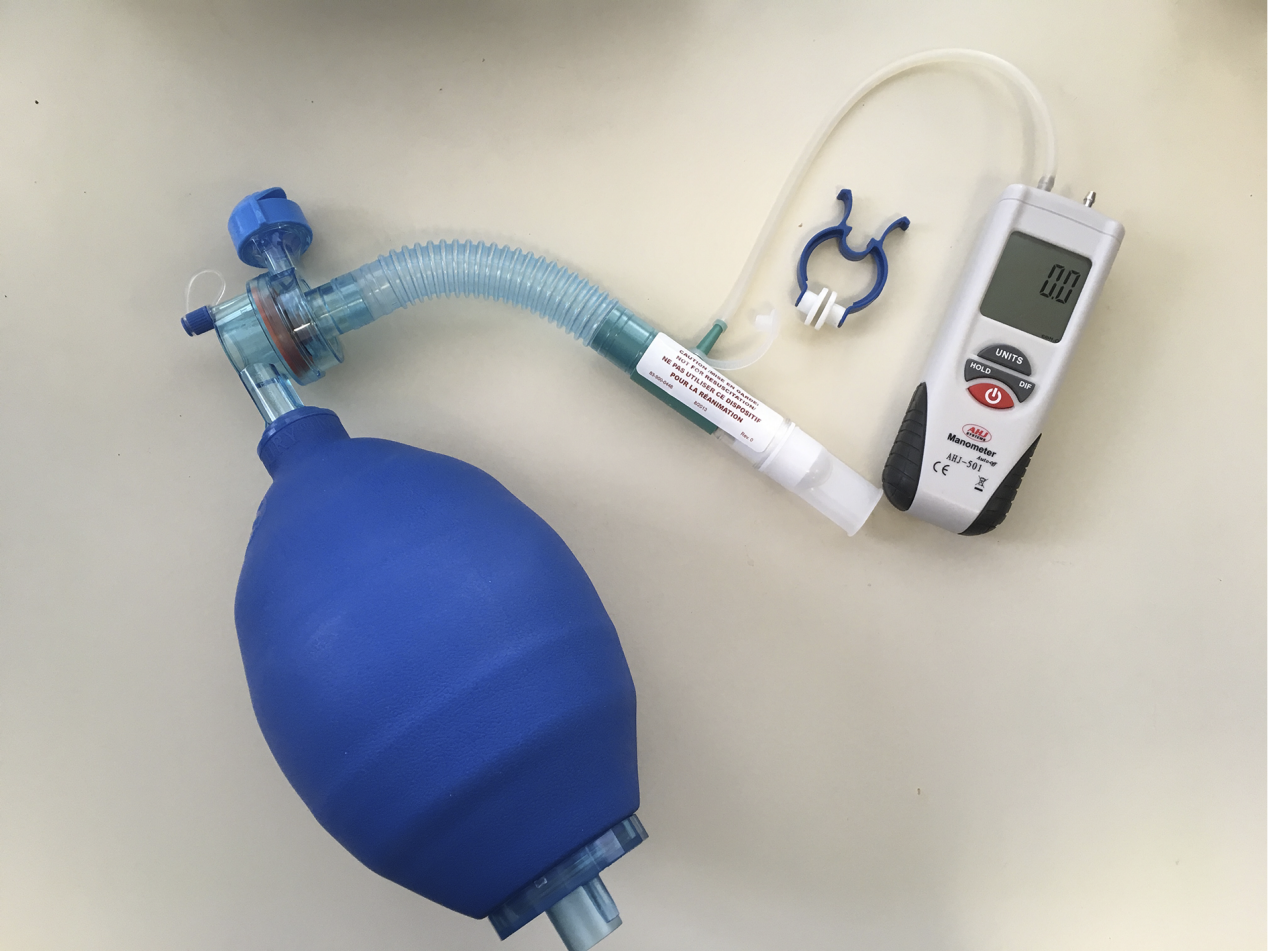
Lung volume recruitment kit, consisting of a manual resuscitation bag, tubing, one-way valve (optional), and mouthpiece (can be interchanged for a mask). The kits may be commercially available such as this one (1.6-L bag, Item number 1034502, Mercury Medical, Florida, USA), or constructed from components available in most hospitals. A digital manometre is attached during clinical assessments in order to check the peak inspiratory pressure reached.

A growing number of consensus statements and disease-specific treatment guidelines include other respiratory therapies as adjuncts to care (15–22). Acknowledging that there is a broad range of adjunctive respiratory therapies, this narrative review will focus on lung volume recruitment (LVR). Lung volume recruitment utilises a resuscitation bag with or without a one-way valve to supplement the volume of air that can be breathed in (15, 23, 24). Consecutive bag compressions deliver positive pressure via a mouthpiece or mask, culminating in an assisted inflation “deep breath” (Figure 1). Performing LVR can improve the expiratory airflow produced during cough (24), potentially enhancing the clearance of mucus. It may be beneficial during acute respiratory tract infections (RTIs) and has also been recommended as a daily prophylactic routine, with the aim of maintaining lung and chest wall “flexibility”, preventing RTIs and slowing respiratory system decline (15–22).

Much of the rationale for using LVR in NMD has been based on clinical reasoning and extrapolation from other patient populations. For example, in the intensive care unit, hyperinflation manoeuvres similar to LVR are performed in intubated patients to inflate areas of under-expanded or collapsed lung and aid sputum clearance (25–27). Whilst more recent studies of LVR in paediatric (28) and adult NMD (23, 29, 30) have provided more direct, patient population-specific data, the evidence base remains thin, and clinical practice is only partially based on research findings.

This narrative review summarises our current understanding of the structural (respiratory muscle weakness, lung volume impairment, etc.) and functional (cough efficacy, breathing compromise during sleep, etc.) consequences of NMDs, in both paediatric and adult disease. The evidence of impact with LVR therapy in NMD is presented, research gaps are identified, and potential future developments in the areas are proposed.

## Respiratory system dysfunction in neuromuscular disease

Clinically, the inability to take a deep breath, as measured by a fall in vital capacity (VC), is a distinctive feature of many NMDs (31). However, despite much of our early understanding of respiratory physiology and normal mechanics of breathing coming from studies on people with spinal cord injury (SCI) or other long-standing and slowly progressive NMDs, there is little research examining the respiratory function and the physiological changes in people with NMD over the course of their disease.

In older children and adults with NMD, restrictive ventilatory impairment is the hallmark of respiratory system involvement, with slow VC, forced vital capacity (FVC), and total lung capacity (TLC) declining as diseases progress (3, 23, 32–34). In those with normal physiology, FVC gradually increases alongside TLC with musculoskeletal growth, reaching a maximum plateau at approximately 20 years of age (35–37).

In children with Duchenne muscular dystrophy (DMD), the FVC peak is reached much earlier, at around age 15 (38). As growth slows and muscle weakness continues to progress, FVC enters a phase of marked decline into early adulthood. When considering % predicted FVC values, there appears to be a linear decline of 4.5%–8.0% predicted per year on average between the ages of 11–22 years (32, 39–42). Treatment with glucocorticosteroid therapy preserves % predicted FVC for longer; however, the rate of decline per year from peak FVC is similar between the steroid-treated and steroid-naïve patients (33, 43, 44). The decline in ALS/MND is much faster, with a loss of 3.1%–3.5% predicted per month (37.2%–42.0% predicted per year) (34, 45), or an overall rate of −1.22 L/year prior to NIV implementation when all phenotypes are considered (7).

In slowly progressive NMDs, the reduction in lung volume and specifically VC reflects a decline in inspiratory capacity (IC) and expiratory reserve volume (ERV) related to inspiratory and expiratory muscle weakness (32). However, whilst VC is highly correlated with muscle strength (23, 46), loss of lung volume is greater than that expected for the degree of muscle weakness alone (46–50). Reduced lung distensibility secondary to microatelectasis and/or changes in the elastic properties of the lungs and chest wall leading to “stiffness of the respiratory system” [i.e., a decrease in total respiratory system compliance (*C*_rs_)] has long been postulated as a factor contributing to lung volume restriction (48, 51). Six studies have investigated *C*_rs_ in people with NMD (Table 1) (23, 29, 48, 51–53), demonstrating that *C*_rs_ is lower in slowly progressive NMDs compared with the healthy control volunteers. In the absence of any longitudinal measures of *C*_rs_ in people with NMD, the strongest evidence that volume loss is related to weakness and chest wall and/or lung tissue stiffness comes from the observation that people with ALS/MND, who experience rapid onset muscle weakness in adult life, have similar degrees of muscle weakness but larger lung volumes and *C*_rs_ than people who have lived with an NMD for decades (23).

**Table 1:** Studies of respiratory system compliance measurements in NMD populations.

Author	Year	Number of participants	Population studied	Findings
Estenne et al. (48)	1983	16 NMD	Chronic SCI, slow NMD	*C*_rs_ lower than controls (mean 0.078 vs. 0.135 L/cmH_2_O)
		20 controls	Healthy volunteers control	*C*_rs_ correlated with TLC and VC in both groups
				No correlation between *C*_CW_ and FRC
Estenne and De Troyer (52)	1986	20 NMD	Chronic SCI	Low *C*_CW_ and *C*_L_ compared with % predicted
				Calculated *C*_rs_ from raw data → mean 0.079 L/cmH_2_O
McCool et al. (51)	1986	14 NMD	Slow NMD, chronic SCI	*C*_rs_ lower than controls (mean 0.075 vs. 0.137 L/cmH_2_O).
		6 controls	Healthy volunteers control	*C*_CW_ and *C*_L_ lower than controls
Scanlon et al. (53)	1989	10 NMD	Acute & chronic SCI	Calculated *C*_rs_ from raw data → mean 0.105 vs. 0.158 L/cmH_2_O
		5 controls	Healthy volunteers control	*C*_L_ lower than controls but *C*_CW_ highly variable
Papastamelos et al. (54)	1996	18 NMD	NMD, aged 3 months to 3.8 years	*C*_CW_ higher in children with NMD than controls (mean 5.2 vs. 2.4 ml/cmH_2_O/kg).
		40 controls	Healthy controls	High chest wall compliance in NMD, comparable with preterm infant data
				*C*_L_ not different between NMD and controls
Molgat-Seon et al. (29)	2017	12 NMD	Slow NMD, chronic SCI	*C*_rs_ lower than controls (mean 0.038 vs. 0.109 L/cmH_2_O)
		12 controls	Healthy volunteers control	
Sheers et al. (23)	2022	80 NMD	Slow NMD (*n* = 53)	*C*_rs_ lower in slow NMD compared with MND (mean 0.033 vs. 0.047 L/cmH_2_O). *C*_rs_ lower than healthy control reference data in both slow NMD and MND groups.
			MND (*n* = 27)	

Note: *C*_rs_ calculated from published raw *C*_L_ and *C*_CW_ values provided in References (52, 53).

NMD, neuromuscular disease; SCI, spinal cord injury; MND, motor neurone disease; *C*_rs_, respiratory system compliance; *C*_CW_, chest wall compliance; *C*_L_, lung compliance; TLC, total lung capacity; VC, vital capacity; FRC, functional residual capacity.

In contrast to the reduced compliance seen in older children and adults, infant-onset NMDs present a unique physiological challenge due in part to the hypercompliance of the chest wall. Though normal for the unaffected infant, young children and infants with NMDs experience a prolonged hypercompliance state and subsequent increased elastic work of breathing that can persist well beyond the first 2 years of life (54). This high compliance state of the thoracic cage coupled with marked muscle weakness and rapid pulmonary and musculoskeletal development frequently results in permanent chest wall deformities and potentially impaired lung growth (1). Eventually, children progress to the low compliance state of older children and adults, which presents a significantly elevated load against which the weakened respiratory muscles must pump (1). Charting this interaction is challenging due to the difficulties of pulmonary function testing in this young cohort. Regardless, the result of these mechanical and muscular challenges can be seen in a steady decline in tidal volume (*V*_T_) during early childhood and frequent challenges with hypoventilation (12), often seen first during periods of sleep.

Sleep is a substantial challenge to effective ventilation, moreso in NMD. Sleep onset is associated with diminished afferent input to the respiratory control centre, in turn altering efferent output; behavioural influences cease with the loss of wakefulness, and metabolic and chemoreceptor sensitivity decrease. Upper airway muscle activity is also reduced during sleep, increasing upper airway resistance. Generalised skeletal muscle atonia during the rapid eye movement (REM) sleep results in inhibition of the intercostal and accessory muscles, thereby leaving the diaphragm as the sole muscle of inspiration (10, 55–57). In addition, the change from the upright to supine position decreases lung volumes, increases the intrathoracic accumulation of blood, and alters the caudal gravitational pull on the intra-abdominal organs, displacing the diaphragm cranially into the ribcage and causing a drop in VC (58).

Infants are particularly vulnerable during sleep due to reduced ventilatory drive, lower baseline resting muscle tone, and increased work of breathing (59). In children under 3 years of age, paradoxical abdominal breathing during the REM sleep is also common, caused by a range of structural and muscular challenges (60). A 3-fold increase in chest wall compliance compared with lung compliance (54) creates negative intrathoracic pressure and indrawing of the rib cage during inspiration (1). Furthermore, a horizontal rib structure, coupled with poor intercostal and accessory muscle activity, means the infant, even when awake, is heavily reliant on the diaphragm (59).

In a person with respiratory muscle weakness, these normal physiological disturbances often have clinical impact. With increasing diaphragm weakness, the fall in supine VC becomes greater (61), and REM-related decrements in activity have more impact. Nocturnal hypoventilation and hypercapnia develop initially, with subsequent daytime hypoventilation and respiratory failure the usual cause of death in many progressive NMDs (2, 6).

Alveolar collapse, mucus plugging, and recurrent RTIs are prevalent respiratory complications (15), and chest wall restriction secondary to scoliosis or kyphoscoliosis also impairs the respiratory function in NMDs. Prior to the widespread use of glucocorticosteroids in DMD, nearly all patients developed scoliosis in the second decade of life (62), but it is currently unclear whether scoliosis is mitigated or simply delayed now that the life expectancy in DMD (63) and other congenital NMDs has increased. Regardless, many people with NMD have restrictive chest wall disease in addition to respiratory muscle weakness.

Involvement of the bulbar muscles can also compromise the respiratory function. Approximately 20%–30% of people with ALS/MND have the bulbar-onset phenotype, and the vast majority of all those with ALS/MND develop bulbar symptoms as the disease progresses (64). Bulbar muscle dysfunction can also occur in SMA, myotonic dystrophy, post-polio syndrome, and in the later stages of DMD (63). Bulbar muscle dysfunction can manifest as some or all of dysphagia, aspiration of oral secretions and/or food, dysarthria, impaired cough, and weak or poorly coordinated movement of the vocal cords (glottis) (65).

Whilst difficulty clearing sputum secondary to changes in airway cilia or mucus rheology is uncommon in NMD, mucus consistency can be affected secondary to aspiration of oral secretions or dehydration. The accumulation and drooling of saliva (sialorrhoea) is reported in almost 50% of people living with ALS/MND and generally attributed to poor saliva control or pooling secondary to weakness and spasticity in the tongue, facial, and pharyngeal muscles (66).

Surprisingly given these potential respiratory complications, the prevalence of respiratory issues in people living with NMD is poorly quantified. A large population-based cohort study conducted in Ontario, Canada, suggested that a third of all people with an NMD may have respiratory system involvement. Thirty-five percent of the 185,586-person cohort were known to a respiratory specialist, 33% had performed respiratory function tests at least once, and 5% were prescribed domiciliary home mechanical ventilation or continuous positive airway pressure (CPAP) for the treatment of sleep-disordered breathing. Including the two-thirds of the population who did not require specialised respiratory care, the individuals with NMD presented to an emergency department 1.6 times every 3 years for respiratory causes, with most of these requiring hospital inpatient admission (1.4 times per 3 years) (67).

## Cough

### Mechanics of cough

Cough is the primary defence mechanism of the respiratory system. It has roles in airway clearance, airway protection, and lung protection by (i) removing sputum, mucus, or foreign particles within the airway, (ii) preventing or minimising aspiration of foreign material into the airway, and (iii) protecting the lungs during a sudden external application of high distending pressure (68).

Two neurophysiologically distinct cough pathways exist: a reflex or laryngeal cough and a tracheobronchial cough. The former is a vagally mediated reflex, activated when the receptors situated in the larynx or proximal trachea are stimulated. These receptors respond primarily to mechanical stimuli, such as aspiration of foreign matter or excessive distension, and hence have a predominantly airway protection role. In contrast, tracheobronchial cough is generally associated with airway clearance. It can be reflex-initiated by mucus or inhaled particles stimulating chemosensitive receptors distal to the larynx in the tracheobronchial mucosa and submucosa (a “spontaneous cough”), or voluntary (a “volitional cough”) (69). Laryngeal and tracheobronchial coughs have different afferent inputs, neural pathways, and efferent outputs (70, 71). In both cases, the motor response is complex, well-coordinated, and involves activation of laryngeal, respiratory, and abdominal muscles, alongside reflexive activation of pelvic sphincters (69).

**Figure 2:**
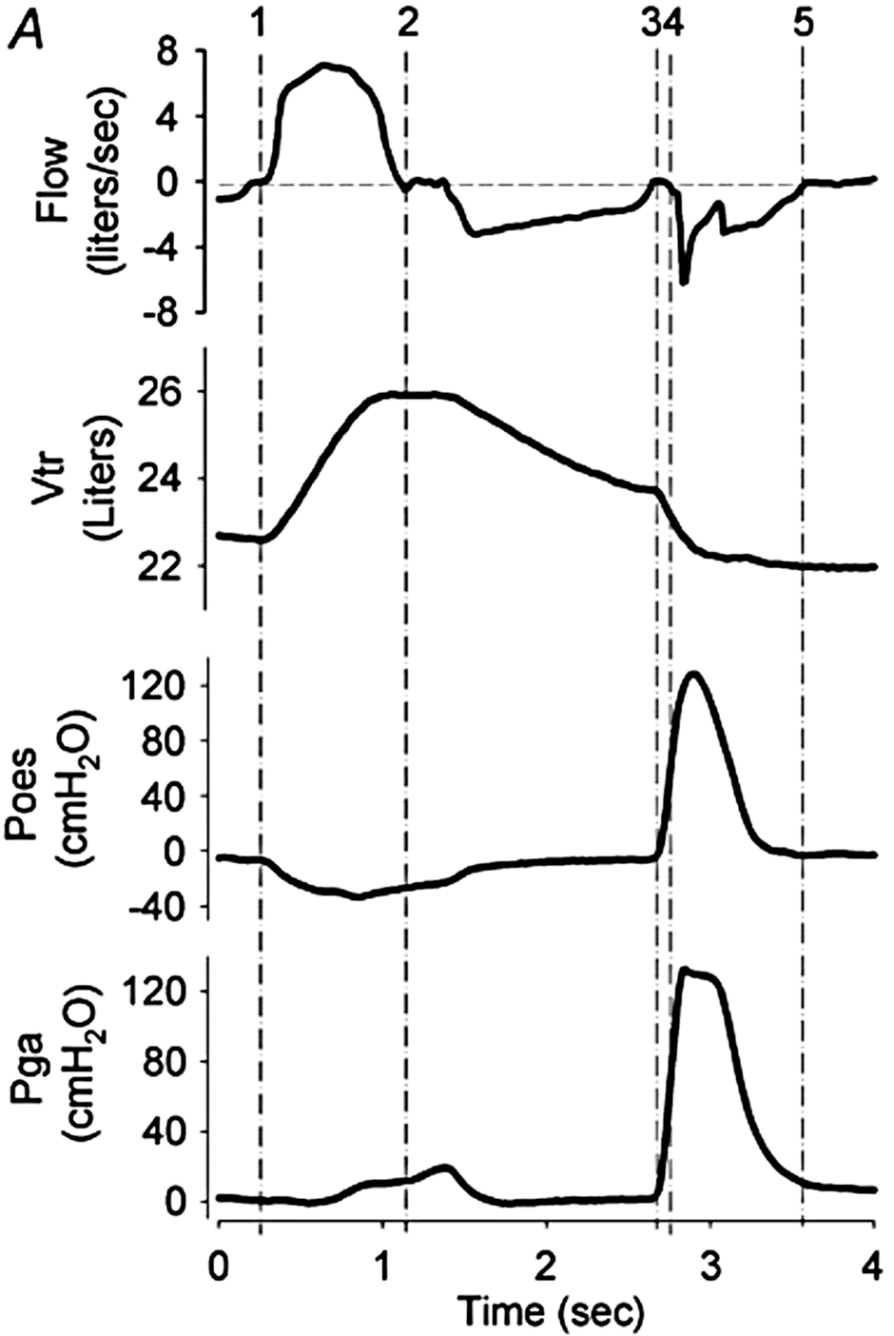
Cough mechanics: flow, volume, and pressure signals during a voluntary tracheobronchial cough. Flow, flow at mouth; volume, trunk volume measured via opto-electronic plethysmography; *P*_oes_, oesophageal pressure (approximation of intrapleural pressure); and *P*_ga_,gastric pressure. Inspiratory phase = vertical lines 1–2, compressive phase = 3­–4, and expiratory phase = 4–5. Note that in this illustration, the period between 2 and 3 signifies exhalation to a predetermined operating volume, as per the experimental protocol. Reproduced with permission from Smith et al. Chest wall dynamics during voluntary and induced cough in healthy volunteers, 2012 (76).

A cough consists of three main phases: inspiratory, compressive, and expiratory (Figure 2) (68, 70, 72). The inspiratory component consists of active abduction of the vocal cords (glottic opening) and inhalation of an adequate volume of air. The size of this inspiration depends on the nature of the cough; during a reflex cough, inspiration is minimal or absent to minimise the risk of a foreign material penetrating deeper into the airway (68). In contrast, during a volitional tracheobronchial cough, the pre-cough inspiratory volume may range from 50% of *V*_T_ to 50% of VC (73) or 85%–90% of TLC (74, 75). These differences in quoted inspiratory volumes possibly reflect differences in instructions, muscle activation/effort, or type of cough (71, 76, 77).

During the compressive phase, the glottis closes and expiratory muscles contract to increase pleural (*P*_pl_) and hence intrathoracic pressure (time points 3 to 4 in Figure 2). This brief closure, lasting 200  ms, is performed by the adductor muscles of the arytenoid cartilages, reinforced by supraglottic structures including the ventricular folds and epiglottis (68). Simultaneously, the expiratory muscles contract isometrically, raising intrathoracic and intra-abdominal pressures. The diaphragm activates in an antagonistic manner to oppose the development of positive pleural pressure. In adults, intrathoracic pressures up to 300 mmHg (400 cmH_2_O) have been described (73). This closing and subsequent abrupt reopening of the glottis is a defining feature of a cough, separating it from a forced expiration. However, the actual significance of this on airway clearance is not clear; expectoration can occur in the absence of glottic closure, for example, during a huff or forced expiration technique or in people with a tracheostomy or laryngectomy (68, 78).

The sudden glottic opening by active vocal cord abduction marks the start of the expiratory phase. Pressure in the central airway (*P*_AW_) falls abruptly to atmospheric, creating a pressure gradient between this central point and the higher *P*_pl_ and alveolar pressure (*P*_A_) developed during the compressive phase. A 30–50 ms burst of supramaximal expiratory flow results, generating a transient “cough spike” with flow rates reaching 10–12 L/s (600–720 L/min) (68). Expiratory flow falls following this cough spike to approximately 3–4 L/s (180–240 L/min); however, the cross-sectional area through which the gas is travelling also decreases, meaning that airflow velocity remains high (due to dynamic airway collapse). Expiratory flows continue for another 200–500 ms, with a concomitant drop in lung volume and *P*_pl_ (Figure 2) (73, 79). The expiratory muscles remain active from the start of the compressive phase through to the completion of this expiratory phase.

### Assessment of cough

The appreciation of cough neurophysiology and mechanics is growing; however, the assessment of cough is not as well developed. The peak expiratory flow rate achieved during a cough (peak cough flow, PCF or cough peak flow, CPF) is the most widely used measure and has been adopted as a surrogate marker of “cough effectiveness”; however, other metrics such as cough volume may be more important in NMD. Although PCF evolved from the measurement of peak expiratory flow (PEF) (80, 81), PCF has not undergone the same scientific scrutiny. In contrast to the assessment of lung volumes or respiratory muscle strength, the European Respiratory Society statement on PCF testing lacks equipment specification details such as device type, resistance and frequency response, or preferred interface (72). Various equipment has been used in published studies, including analogue or digital peak flow metres (82–89), handheld spirometres (90), and the gold-standard measurement device, a pneumotachograph (23, 91–95); yet, inter-device agreement has not been adequately determined.

Peak flow metres, primarily used for PEF measurement in asthma management, are inexpensive, readily available devices that have been repurposed by some clinicians and researchers to measure PCF. However, considerable PEF measurement error has been demonstrated (96–99), depending on the brand tested (limits of agreement −139–109 L/min) (97). A cough presents an even greater challenge to measure than a forced expiration; devices must have adequate dynamic response characteristics to capture the rapid acceleration and transient supramaximal peak flow of the manoeuvre (100). Some studies have reported comparable PCF values between pneumotachograph and peak flow metres (101, 102) whereas other authors have demonstrated considerable disagreement, with mean bias up to 56 L/min (limits of agreement −26–138 L/min) (103–105), or above a flow rate of 270 L/min (91). Similarly, the interface used may effect PCF measurement. Spirometric values obtained with a mouthpiece compared with an oronasal mask are not interchangeable (106), and a small study in healthy young adults suggests that using a mouthpiece yields higher PCF values compared with a facemask (107).

In infants, technical challenges and lack of normative data mean that the value of respiratory function tests in general remains debatable in clinical management. Spirometry testing can be accurate in pre-school children with appropriate environment, equipment, and training (108), though the reliable and consistent performance of spirometry is usually considered to be achieved around 5–6 years of age (109). Despite this, PCF was observed to be random in children with DMD and did not correlate with markers of disease severity (ambulation, age, and steroid use), unlike FVC and TLC (110). As such spirometry measures such as FVC and PEF are likely to be better surrogate measures of cough effectiveness in children with NMDs (111).

Clinical interpretation of any test requires knowledge of “normal” values, within-subject repeatability and reproducibility, but these have not been satisfactorily established for PCF. Reported mean values in healthy adult participants vary widely (71, 74, 76, 91, 95, 101, 112–124), with one study reporting reference values in children from Italy (89). Lower PCF values have been reported in people with NMD compared with healthy control volunteers (74, 91, 95, 101, 118, 119), with one large population study observing a mean PCF value of 264 ± 108 L/min from 155 stable participants with NMD (92). The studies investigating the repeatability of PCF using a pneumotachograph have demonstrated individual coefficients of variation ranging from 8.7% to 20.3% (117, 125), with a single reading determined to be 76% reliable (117).

Despite these issues, PCF is likely to be the most common cough measure in clinical practice. The latest European Respiratory Society statement on respiratory muscle testing includes PCF and states it “estimates the effectiveness of mucus clearance and expiratory muscle function” (72). Testing instructions (“maximal cough after complete inhalation”), number of trials (“3–6 manoeuvres”), and acceptability of manoeuvre criteria (“<5% variability”) are briefly described (72), although it should be noted that these are based on the method of one study (91) rather than repeatability and reproducibility data.

The clinical significance of PCF is unclear; although PCF is commonly interpreted as a marker of cough effectiveness, few studies have related this to difficulty clearing secretions or expectorating sputum. To our knowledge, no studies have directly examined the relationship between PCF and airway clearance in people with NMD. In people with obstructive airways disease, no correlation is observed between radioaerosol clearance of mucus and the PCF achieved (126, 127). Using mathematical simulations applied to a three-dimensional human airway reconstruction model, changing PCF appears to have minimal effect on mucus clearance under normal and low mucus viscosity conditions. Increasing cough expired volume significantly improved mucus clearance (128), and may be a more clinically relevant cough metric compared with PCF. Supporting this hypothesis, cough volume can be used to detect upper airway collapse during mechanical-assisted coughing in people with NMD, whereas PCF continues to increase despite airway closure (129).

Nonetheless, in the NMD field, a threshold of 160 L/min has been reported as the PCF required for effective airway clearance (18, 62), based on the findings of Bach and Saporito (86). This cohort study of adult patients managed at a single ventilator weaning unit aimed to explore the parameters that predicted successful extubation or tracheostomy decannulation. They observed that 32 out of 37 participants achieved this outcome, all of whom recorded a PCF >160 L/min (with assistance) (86). However, the significant limitations of this study cast doubt on the validity of this cut-off, including the small single-centre sample, heterogeneous non-NMD diagnoses, omission of potential confounding factors on extubation success, poorly described statistical analyses, and no prospective validation. Moreover, although interpreted as the PCF needed for “effective airway clearance”, the source data did not objectively measure airway clearance.

Indeed, most studies concerning cough in people with NMD have not examined the ability of cough to clear secretions, instead focusing on the relationship between PCF and other respiratory function tests. One of the largest studies tested 155 consecutive patients undergoing routine clinical review and observed a correlation between PCF and VC (*r*^2 ^= 0.58). Stepwise regression identified that the IC component contributed 44% of the variance (92). Another large cohort of 179 stable participants with NMD found that a VC >1.18 L or maximal expiratory pressure (MEP) > 24 cmH_2_O predicted the ability to achieve a PCF >180 L/min (90). The associations between measured PCF and VC, pre-cough inspired volume, maximal inspiratory pressure (MIP), and/or MEP have also been reported (74, 83, 95, 119, 122, 130–134).

Only a small body of research from Sancho and colleagues has functionally defined cough by its ability to effectively clear secretions. A year-long prospective cohort study of patients with ALS/MND referred to a specialist centre aimed to identify the factors when well that may predict who has a clinically defined ineffective cough when sick (94). A total of 40 of the 53 participants subsequently had an acute RTI, during which time 35% could effectively clear secretions. Those who could not clear secretions had worse lung function at baseline. Moreover, PCF, PCF/peak velocity time, and bulbar score were the most accurate predictors of who would have an ineffective cough when unwell (proposed PCF cut-off value <255 L/min) (94). In a subsequent prospective cohort of participants with ALS admitted to the same centre with an acute RTI, 93% (44 of 48 participants) could not clear secretions without assistance. In agreement with the earlier study, the participants with an ineffective cough had poorer FVC, PCF, MIP, and MEP compared with the four patients who could cough effectively (proposed PCF cut-off value <166 L/min) (135). Whilst these data imply an association between FVC, PCF, and ability to clear secretions, both studies omit the patients who did not have an RTI from the analysis; whether they too had poor respiratory function but remained well and could effectively clear secretions is unknown.

Collectively, the studies in people with NMD imply that VC, MIP, MEP, and/or bulbar function are associated with cough. Broadly, these measures reflect higher lung volume, better diaphragm and abdominal muscle strength, and glottic closure, each important for the generation of intrathoracic pressure. There are no data examining the correlation between these factors and pleural pressure (*P*_pl_) in people with NMD; however, based on first principles, it is possible that variables such as lung volume are linked to *P*_pl_ and hence an “effective cough” in this population. However, the reverse is also true, in that high *P*_pl_ can also be generated in the absence of high lung volumes. A study conducted in people with tetraplegia demonstrated that whilst abdominal muscle stimulation and glottic closure during a cough manoeuvre produced the highest oesophageal (*P*_oes_), gastric pressure (*P*_ga_) and PCF with concomitant airflow limitation in all participants, an un-stimulated cough (i.e., little or no expiratory muscle activity) against a closed glottis still achieved flow limitation in half the group (136).

Earlier studies have also demonstrated that people with respiratory muscle weakness can generate dynamic compression of intrathoracic airways, even at markedly subnormal peak *P*_pl_ (137). Whilst the glottis is one determinant, people with lower respiratory system compliance (*C*_rs_) can achieve an adequate *P*_pl_ due to the effect of higher chest wall and/or lung recoil pressures (138). Thus, people with NMD may not have normal cough mechanics but may have a more effective cough than conventionally thought. This view has been shared by others, with Young and colleagues stating over 30 years ago: “For almost 150 years the importance of high flow rates and glottic closure have dominated thinking on the mechanics of cough … … It is equally important to consider that cough can be productive without glottic closure and with low flow rates” (139).

## Respiratory tract infections in neuromuscular disease

People with NMD are at a greater risk of developing atelectasis, pneumonia, or RTIs (15–17, 20, 62, 63, 140, 141), and acute illness can exacerbate underlying respiratory muscle weakness, perpetuating respiratory dysfunction. Statistically significant falls in MIP and MEP (24% and 29%, respectively, in NMD, and 10% and 12% in health adults) typically accompany viral upper RTIs in healthy adults and people with NMD, lasting up to 14 days post-illness onset (142, 143). Shortness of breath, acute hypercapnia, and decline in VC are observed in people with NMD during RTI (143).

Reported RTI rates vary widely, for example, from 9% to 75% in people with ALS/MND (23, 88, 94, 144–146). The variance in rates may be explained by differences in study methodology (retrospective chart review vs. prospective data), RTI definition (viral, bacterial, community-acquired, etc.), observation period (12–50 months), and usual respiratory care provided. The best population data are from the large Canadian cohort by Rose and colleagues of all NMD diagnoses. They demonstrated a respiratory admission rate of 1.4 times every 3 years/person, and further, that people with ALS/MND were more than four times more likely to present to the emergency department than those with slowly progressive NMD (67).

Most data on infection rate are however from single centres and/or retrospectively collected. For example, 672 ventilation users with NMD were asked to recall prior episodes of pneumonia or hospitalisation. Unfortunately, these data are difficult to analyse because they were split into 18 treatment categories over different time periods, and the overall rates were not well summarised (147). Other retrospective studies from the same authors have compared episode and hospitalisation rates before and after the introduction of a home-based respiratory monitoring protocol; however, these data thus represent a biased sample of only those patients with a history of RTI (148, 149).

Within a SCI population, retrospective chart review at a single centre identified that 23% of patients over a 4-year period had a diagnosis of pneumonia, with FVC and MIP being significantly lower in those who had (150). In a sample of 37 patients with DMD presenting for routine assessment, over half (59%) reported an RTI in the preceding year (130). Using a similar conservative definition of “have you suffered a chest infection in the past year?”, Dohna-Schwake and colleagues reported an average RTI rate of 0.84 ± 0.94 episodes/patient/year. Of the 46 paediatric participants with NMD, 22 patients (48%) reported a “severe chest infection” defined as an admission after the age of 2 years, thereby including any episode within a 4–18-year period (participant mean age = 12.7 years).

A similar incidence has been reported in 80 adults with heterogeneous NMDs; 43% of the participants recalled “a chest infection that required antibiotic treatment within the past year”, an incidence of 0.60 episodes/participant/year. People with slowly progressive NMDs had higher RTI rates than those with ALS/MND (53% compared with 22%), possibly due to lower lung volumes, as VC was the only respiratory marker found to be associated with RTI (23). This incidence in people with ALS/MND is much lower than the 75% reported in the year-long prospective cohort by Sancho and colleagues (94), but similar to a single-centre prospective cohort (30%) from Ireland (146), and may reflect differences in NIV use and other ALS/MND care.

Given that respiratory complications are the primary cause of discomfort and death in people with NMD, authors have sought to predict those at risk of developing an RTI. In the paediatric cohort, a VC <1.1 L or PCF <160 L/min were able to distinguish those who had a severe RTI in the past or not (131). In DMD, the observation that none of the 46 patients who started a respiratory management protocol when their PCF fell below 270 L/min developed an RTI (149) has been interpreted as the PCF threshold needed to prevent hospital admission and reduce the risk of pneumonia. Moreover, clinical care guidelines recommend assisted coughing strategies such as a mechanical insufflation–exsufflation (MI-E) be implemented once PCF <270 L/min (17, 18, 20, 62, 63, 140, 141, 151).

However, these thresholds have been derived from retrospective data and not validated prospectively. The respiratory function has been measured post RTI (131); hence, poorer lung function may be a result of a previous infection rather than the cause as has been implied. The studies have excluded the patients without RTI (149), which introduces significant selection and reporting bias, limiting generalisability. Furthermore, baseline PCF values in young children with NMDs are below these adult-specific values suggested for starting assisted cough techniques (152), and hence it is unclear if these thresholds are useful predictors in paediatric NMDs.

In a recent adult cohort, these threshold values were unable to discriminate the participants who had an RTI history or not, with thresholds of VC <1.1 L or PCF <270 L/min correctly classifying only 61% and 50% of the group, respectively (23). These findings cast doubt on the sensitivity and specificity of VC or PCF to predict RTI risk, and require replication given the widespread use of these thresholds in clinical practice. Prospective cohort studies and comprehensive “big” data spanning paediatric and adult populations are required to better determine the risk of acquiring an RTI in those living with an NMD.

## Respiratory management in neuromuscular disease

Given the relationship between poor lung function, morbidity, and mortality, multidisciplinary respiratory care is considered vital for people with respiratory dysfunction secondary to NMD. Monitoring function, initiating therapies at the appropriate time, ongoing assessment and titration of treatments, responding to acute illness, and providing suitable end-of-life care are all important clinical care objectives.

Ventilatory support is an established and key treatment for hypoventilation and chronic ventilatory failure; NIV has a significant impact on survival when nocturnal hypoventilation is present and is recommended in clinical care guidelines (18–21, 141). Many review articles have been published that synthesise the evidence (4, 5, 8–10), and other sections of this Frontiers Special Topic discuss role, criteria, timing of commencing NIV, and other still-debated issues.

Adjunctive respiratory management of people with NMD is less clearly defined. Summarising the evidence of efficacy is challenging as the research is of a lesser quality, and various techniques and dosages are employed (24). Whilst there is a small body of research investigating inspiratory and/or expiratory respiratory muscle training in this population (153–159), most clinical and research activity is focussed on airway clearance techniques (ACTs).

Airway clearance techniques are therapies that aim to improve cough effectiveness, mobilise mucus, and increase lung volume (Table 2). A consensus meeting convened in 2017 by the European Neuromuscular Centre discussed the “clearance” function of ACTs, categorising techniques as peripheral ACTs (techniques that improve ventilation, loosen secretions, and enhance mucus transport from peripheral to central airways), or proximal ACTs (techniques that augment cough). The authors concluded that ACTs are beneficial for mucus clearance and cough augmentation; however, the effect of ACTs on lung volume was not discussed in detail (15, 16). A narrative review extended this work by considering the volume augmentation effects of these techniques (24).

**Table 2:** Taxonomy for airway clearance techniques.

Objective	Method	Technique	Type
Cough augmentation	Inspiratory assistance	Single or stacked-breath methods	Assisted inflation therapies (see below)
	Expiratory assistance	Manual	Manually assisted cough (MAC)
		Mechanical	ME of MI-E
	Combined assistance	Manual	Inflation + MAC
		Mechanical	Mechanical insufflation-exsufflation device (MI-E)
Volume augmentation	Assisted inflation	Stacked-breath methods	Glossopharyngeal breathing
			LVR: manual resuscitation bag
			LVR: VCV-NIV
		Single-breath methods	Pressure-limited NIV
			Inspiratory positive pressure breathing device (IPPB)
			MI of MI-E
Sputum clearance	Peripheral ACT/sputum mobilising	Manual	Manual techniques (positioning, percussion, vibrations)
			Chest wall strapping
		Mechanical	Intrapulmonary percussive ventilation
			High frequency chest wall oscillation
	Proximal ACT/cough augmentation	Inspiratory, expiratory, or combined methods	See above

MAC, manually assisted cough; ME, mechanical exsufflation; MI-E, mechanical insufflation-exsufflation; LVR, lung volume recruitment; NIV, non-invasive ventilation; VCV-NIV, volume-limited mode (i.e., volume-controlled ventilation) of non-invasive ventilation; IPPB, inspiratory positive pressure breathing; MI, mechanical insufflation.

Airway clearance techniques have traditionally been employed to augment cough or to reinflate areas of collapsed lung, improve alveolar ventilation, and mobilise mucus from the peripheral and central airways during sputum encumbrance or acute respiratory illness (160). More recently, it has also been recommended that they be performed routinely, although evidence for long-term clinical benefits is limited (17–22). This shift, from initiating treatments only when there is a clear indication to a proactive approach of introducing therapies earlier, has occurred in parallel with advances in the medical management and prognosis of many NMDs (19, 161). Many patients are living longer with a chronic disease, with therapy aims reflecting this chronic disease management paradigm.

“Assisted inflation” ACTs increase inspiratory volume above the spontaneous unassisted IC, thereby hyperinflating the lungs and mobilising the chest wall. Volume recruitment techniques [namely manual hyperinflation or ventilator hyperinflation (160, 162)] do increase lung volume, sputum clearance, and *C*_rs_ in intubated and ventilated patients (25–27). Multiple authors have speculated that augmenting lung volume in people with respiratory muscle weakness or restrictive chest wall disease when well may improve long-term respiratory system “flexibility” and prevent deleterious respiratory sequelae (19, 20, 39, 62, 163). Averting RTIs, particularly in patients with a history of recurrent episodes, may prevent secondary parenchymal pathology or further lung function decline. Extrapolating from data in NIV, it has also been postulated that slowing lung volume decline may have a beneficial effect on respiratory symptoms, complications, and potentially even survival.

Whilst there is clinical rationale and biological plausibility to hypothesise that assisted inflation therapies may maintain or improve lung volumes, *C*_rs_, and potentially slow the decline in the respiratory function, there is a paucity of supporting research. Lung volume recruitment using a manual resuscitation bag is one method of assisting inflation that has wide clinical appeal. It is a simple therapy that uses inexpensive and widely available equipment, thereby having advantages over other assisted inflation techniques that require a machine to deliver the driving pressure. The remainder of this narrative review will describe LVR and the current evidence base for this therapy.

## Lung volume recruitment

**Figure 3:**
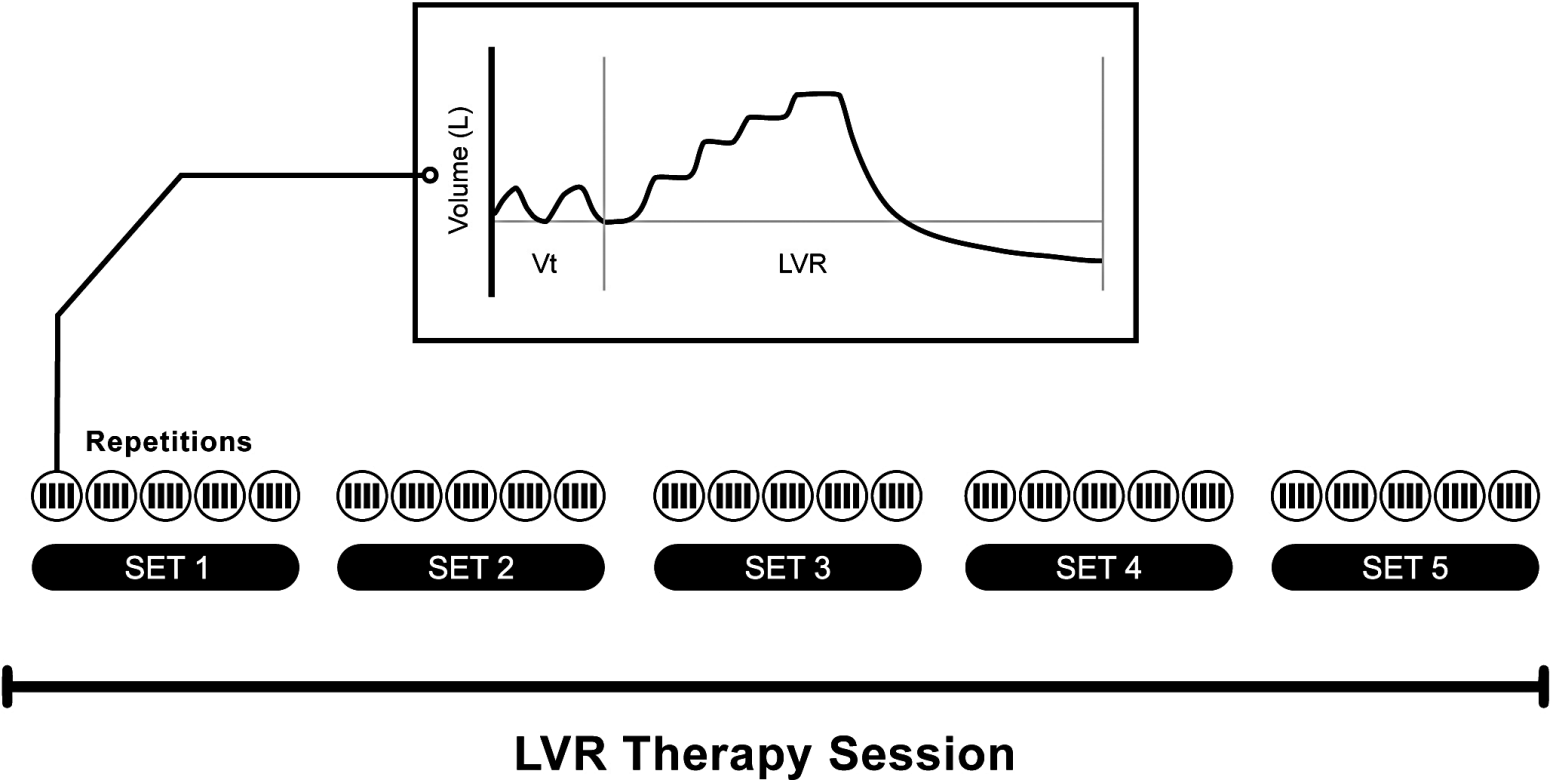
Example of a lung volume recruitment therapy session, as may be prescribed for regular volume recruitment. This schematic illustrates a session comprising five sets (or cycles) of five maximal inflation repetitions. The number of compressions required to reach the maximum tolerable inflation capacity is determined for each individual based on chest wall excursion, patient comfort, and, in some clinical guideline**s,** the pre-defined upper pressure limit (e.g., 40 cmH_2_O) (15). The trace is indicative of the volume signal observed during tidal volume breathing followed by a single maximal inflation repetition (as captured by a pneumotachograph). Note the “stepped” pattern of inspiration during the LVR manouevre.

Lung volume recruitment, commonly known as “breath stacking”, is a stacked-breath assisted inflation technique (see Table 2 for a full list of assisted inflation technologies). Regardless of the source of the driving pressure, consecutive compressions or insufflations are performed, without exhaling in between, until the maximum, tolerable inflation capacity is reached. This volume, equivalent to an externally assisted IC, is termed the maximum or lung insufflation capacity (MIC or LIC), depending on whether glottic control is necessary for the assisted inflation (MIC) or not (LIC) (15, 164). When employed for cough augmentation, exhalation from MIC or LIC is forced (i.e., cough or huff) and may be combined with expiratory assistance such as a manually assisted cough (MAC). In contrast, if the primary aim is volume recruitment, then assisted inflations are followed by passive exhalation to functional residual capacity (FRC) (Figure 3).

**Figure 4:**
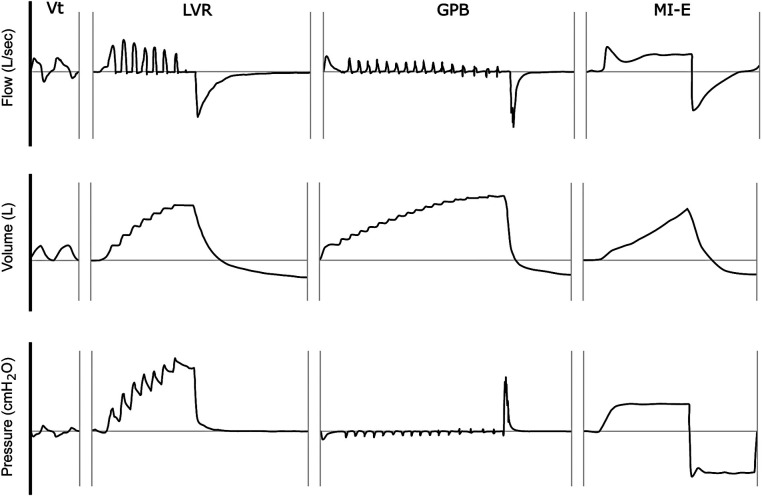
Stacked vs. single-breath methods of assisted inflation. This schematic illustration depicts the flow, volume, and mouth pressure signals observed (as captured by a pneumotachograph) during (i) tidal volume (*V*_t_) breathing, (ii) single maximal inflation repetition (LVR), (iii) glossopharyngeal breathing (GPB), and (iv) mechanical insufflation (MI) of a mechanical insufflation-exsufflation (MI-E) device. The horizontal axis represents time (ms). Note the “stepped” pattern of inspiration during the LVR and GPB manoeuvres, in contrast to the single inflation pattern of MI.

The “stacking” of breaths distinguishes LVR from other methods of assisted inflation (Table 2). Stacked-breath methods are characterised by brief plateaus of zero flow between consecutive insufflations, whereas single-breath methods reach LIC with a single insufflation (Figure 4). All methods can be delivered via a mouthpiece or oronasal mask. If the latter is used, intact bulbar function is theoretically not required for single-breath methods, as the devices can be set to deliver a volume that exceeds unassisted spontaneous IC. However, glottic control is necessary to perform glossopharyngeal breathing, LVR with a volume-limited NIV machine, or LVR with a manual resuscitation bag without a one-way valve, as the vocal cords must repeatedly adduct to hold the insufflated volume(s) until MIC is reached. Commercially available LVR kits contain a one-way valve, thereby avoiding the need for adequate bulbar function, and are used to achieve LIC (Figure 1).

A proportion of children and young people with NMDs are unable to participate fully in some LVR techniques due to cognitive, developmental, or behavioural limitations, and alternative techniques have been proposed to augment lung volume in cohorts unable to follow commands (165–167). “Involuntary breath stacking” using a facemask and one-way valve alone is another method described by Jenkins et al. This technique achieves inflation volumes similar to “voluntary breath stacking” (spontaneous breath stacking with glottic control) but lower than “supported breath stacking” using an LVR kit with a one-way valve (167).

An advantage of LVR over the other methods is its availability. The equipment is lightweight, portable, inexpensive (approximately €25), and comes ready-assembled. Alternatively, a standard manual resuscitation bag found in any hospital can be employed. Single-breath methods of inflation require a machine to deliver the driving pressure; if patients are not using NIV at home, then machine provision incurs an additional cost. If the person is an existing NIV user, their ventilator could be utilised, but not all machines are capable of performing volume-limited ventilation. Pressure-limited bilevel devices are the most commonly prescribed domiciliary NIV machines and can deliver LVR; however, this requires the users to frequently change ventilator settings back and forth from the nocturnally prescribed pressures to the assisted inflation settings. Inspiratory positive pressure breathing (IPPB) devices or MI-E machines avoid this risk, as they are used for the single purpose of hyperinflating and are fully programmable; however, they are expensive (approximately €6,000), heavier, and less portable. Whilst these machines may be available or funded in some jurisdictions, their cost is prohibitive in many other environments. Furthermore, they arguably require more specialised skills to set up and titrate (168, 169), and may require ongoing technical support and service—additional barriers to access.

In addition to equipment used or role of the glottis, there are other minor differences in how assisted inflation therapies are clinically applied, although research evaluating these variations is limited. No research has been conducted examining the effect of starting volume on MIC or LIC; some studies commence insufflations *after* an active breath to spontaneous IC (84, 133, 163, 170, 171), whereas other authors either start at FRC or have not defined the starting volume (39, 40, 85, 88, 90, 164, 172–178). A single study has reported larger volumes obtained when the participants actively inhaled and exhaled with a MI-E device compared with passively accepting the pressures delivered; however, this finding has not been replicated (179).

Although rare, the side effects and possible complications have been reported with LVR, glossopharyngeal breathing, and MI-E. The qualitative analysis of the participants with cervical SCI who had participated in an 8-week study of glossopharyngeal breathing described transient episodes of dizziness, fainting, loss of vision, tingling, and bloating (180). Syncope has also been reported with LVR (28), as has mild chest discomfort, presumably from stretching of the musculoskeletal system of the thoracic cage (28). Abdominal distension and nausea/retching are other potential side effects of hyperinflation, reported with MI-E use (88, 181). These aforementioned symptoms usually subside with practice or modification of the technique/s (28, 88, 180, 181). Of greater concern is the potential for significant fluctuations in blood pressure (182, 183), bradyarrhythmias or transient asystole (182), and pneumothorax (184–187). The incidence of serious side effects is not well quantified, and clinicians recommending LVR therapies should balance the potential for harm with the potential for benefit.

## Effect of LVR on volume and PCF during the manoeuvre

The aim of assisted inflation techniques is to hyperinflate the lungs to a maximum, safe, and tolerable volume that is higher than the own spontaneous IC of the person. Broadly, assisted inflation therapy has consistently demonstrated that a LIC or MIC greater than VC can be obtained in more than 90% of the selected participants (173, 174, 188), and that a cough initiated from this augmented volume can increase the measured PCF (Table 3). In adults with NMD naïve to LVR, 95% of the participants were able to achieve a LIC at least 10% larger than VC within a single visit (23). On average, an additional 0.42 ± 0.50 L of air (37% ± 46% of baseline VC) was able to be stacked during the first attempt. This increased following a short session of LVR, which the authors attributed to improved technique in their naïve population (23).

**Table 3:** Studies examining volume and cough augmentation achieved during assisted inflation techniques, in medically stable participants with NMD.

Author/s	Year	Number of participants	Population	Trial design	Hyperinflation therapy	Comparison b/w methods made?	Results
Bach (85)	1993	21	Slow NMD	Prospective	LVR with VCV-NIV MI-E	Yes	MIC > VC PCF_MI-E _> PCF_MIC+MAC _> PCF_MIC _> PCF
Kang and Bach (133)	2000	108	MND Slow NMD	Case series	LVR with bag *or* LVR with VCV-NIV	No	90/108 patients: MIC > VC PCF_MIC+MAC _> PCF
Sivasothy et al. (118)	2001	12	Slow NMD	Prospective Randomised order	MAC MI with MI-E MI + MAC	Yes	IC not measured PCF_MI+MAC _= PCF_MAC_ > PCF_MI_ = PCF
Chatwin et al. (95)	2003	22	Slow NMD	Prospective Randomised order	MAC NIV ME of MI-E MI-E	Yes	IC not measured PCF_ME _= PCF_MI-E _> PCF
Mustfa et al. (119)	2003	47	MND	Prospective Randomised order	MAC ME of MI-E MI of MI-E MI-E	Yes	IC not measured PCF_MAC_ = PCF_ME _= PCF_MI-E _> PCF
Sancho et al. (173)	2004	26	MND	Prospective Order not randomised	LVR + MAC MI-E + MAC	Yes	24/26 patients: MIC > VC
Kang et al. (170)	2005	71	DMD	Prospective	LVR MAC LVR + MAC	Yes	MIC > VC PCF_MIC+MAC _> PCF_MAC _= PCF_MIC_ > PCF
Trebbia et al. (92)	2005	10	Slow NMD	Prospective Randomised order	MAC IPPB IPPB + MAC	Yes	LIC with IPPB + MAC > IPPB alone PCF_IPPB+MAC_ > PCF_IPPB_ = PCF_MAC _> PCF
Dohna-Schwake et al. (188)	2006	29	NMD	Prospective	IPPB	No—single technique	28/29 patients: LIC > VC 27/29 patients: PCF_IPPB+MAC _> PCF
Kang (134)	2006	40	SCI	Prospective	LVR MAC LVR + MAC	Yes	MIC > VC PCF_MIC+MAC _> PCF_MAC _> PCF_MIC_ > PCF
Bach et al. (174)	2007	78	DMD	Case series	LVR with bag *or* LVR with VCV-NIV GPB in subgroup	Yes	74/78 patients: MIC > VC PCF_MIC+MAC _> PCF
Bach et al. (164)	2008	282	MND Slow NMD	Case series	LVR with bag *or* LVR with VCV-NIV LVR with bag & valve	Between two techniques only	LIC > MIC > VC PCF_MIC _> PCF Did not measure PCF_LIC_
Ishikawa et al. (176)	2008	61	DMD	Prospective Randomised order	LVR MAC LVR + MAC	Yes	IC not measured PCF_LVR+MAC_ > PCF_LVR_ > PCF_MAC _> PCF
Toussaint et al. (90)	2009	179	Slow NMD	Prospective	MAC LVR with VCV-NIV LVR + MAC	Yes	IC not measured PCF_LVR+MAC_ > PCF_LVR_ = PCF_MAC _> PCF
Brito et al. (132)	2009	28	DMD	Prospective Randomised order	MAC LVR LVR + MAC	Yes	IC not measured PCF_LVR+MAC_ > PCF_LVR_ = PCF_MAC _> PCF
Senent et al. (93)	2011	16	MND	Prospective Randomised order	MAC LVR + MAC NIV + MAC MI-E	Yes	IC not measured PCF_LVR+MAC_ = PCF_NIV+MAC_ = PCF_MI-E _> PCF_MAC _> PCF
Bianchi et al. (189)	2014	18	Slow NMD	Prospective	MAC LVR GPB Self-thrust LVR + MAC GPB + MAC GPB + self-thrust	Yes	IC not measured PCF assisted techniques > PCF unassisted. No difference between techniques
Lacombe et al. (190)	2014	18	Slow NMD	Prospective Randomised order	IPPB + MAC MI-E MI-E + MAC	Yes	LIC achieved with all three conditions greater than unassisted PCF_IPPB+MAC_ > PCF_MI-E+MAC_ > PCF_MI-E _> PCF
Mellies and Goebel (82)	2014	29	Slow NMD	Prospective Randomised to group	IPPB Lung insufflation assist manoeuvre (LIAM)^a^	No	LIC_IPPB_ > VC, and LIC_LIAM_ > VC Optimal LIC to achieve highest assisted PCF = 89%–91% of max LIC
Torres-Castro et al. (84)	2014	15	SCI	Prospective Randomised order	MAC LVR LVR + MAC	Yes	IC not measured PCF_LVR+MAC_ > PCF_LVR_ = PCF_MAC _> PCF
Toussaint et al. (191)	2016	52	DMD	Prospective Randomised to group	LVR with bag LVR with VCV-NIV	Yes	MIC_bag_ = MIC_VCV-NIV_ PCF_bag _= PCF_VCV-NIV_
Santos et al. (179)	2017	47	Slow NMD	Prospective	Passive MI-E Actively assisted MI-E	Yes	LIC_active_ > LIC_passive _> Spontaneous IC
Sarmento et al. (192)	2017	12 MND 12 Controls	MND	Cross-sectional Matched pairs	LVR	N/A: Comparison b/w MND and healthy controls	PCF_LVR _> PCF in MND Increase in IC (chest wall and abdominal compartmental volumes) via OEP during LVR
Nygren-Bonnier et al. (183)	2018	10	SCI	Prospective	Glossopharyngeal breathing	No—single technique	MIC > VC. Also increased TLC, HR during manoeuvre, decreased MAP
Kikuchi et al. (105)	2018	12	DMD	Prospective Randomised order	MAC LVR LVR + MAC MI-E MI-E + MAC	Yes	LIC not measured, only PCFs PCF_MI-E+MAC _= PCF_MI-E _= PCF_LVR+MAC_ > PCF_LVR_ > PCF_MAC _> PCF
Iskandar et al. (193)	2019	8	DMD	Prospective	MAC LVR LVR + MAC	Yes	LIC not measured, only PCFs PCF_LVR+MAC_ = PCF_LVR_ = PCF_MAC _> PCF
Del Amo Castrillo et al. (194)	2019	20	Slow NMD	Prospective Randomised order	LVR with VCV-NIV (MIC) Volumetric cough mode (VCM)^b^ (LIC)	Yes	MIC vs. LIC not statistically different PCF_LIC_ > PCF_MIC_ > PCF Comfort and perceived cough effectiveness similar

NMD, neuromuscular disease; MND, motor neurone disease; DMD, Duchenne muscular dystrophy; SCI, spinal cord injury; LVR, lung volume recruitment; VCV-NIV, volume-limited non-invasive ventilation; MAC, manually assisted cough; NIV, non-invasive ventilation; MI, mechanical insufflation; MI-E, mechanical insufflation-exsufflation; ME, mechanical exsufflation; IPPB, inspiratory positive pressure breathing; GPB, glossopharyngeal breathing; MIC, maximal insufflation capacity; VC, vital capacity; PCF, peak cough flow; LIC, lung insufflation capacity; IC, inflation capacity; TLC, total lung capacity; HR, heart rate; MAP, mean arterial blood pressure; OEP, opto-electronic plethysmography.

^a^ VENTIlogic LS ventilator mode: pressure-controlled manoeuvre with preset insufflation time and pressure plateau phase.

^b^ Astral 150 (Resmed) ventilator mode: volume-controlled hyperinflation breath titrated to achieve LIC, with maximum allowed 500% of baseline *V*_T_ or PIP 50 cmH_2_O.

The shaded rows highlight studies that compared degree of inflation volume (i.e., LIC or MIC) between techniques.

The studies evaluating the degree of volume recruitment achieved during the manoeuvre suggest that no one method of hyperinflation is superior overall. The proportion of the participants with DMD who could successfully employ LVR using a resuscitation bag was similar to the success rate using volume-limited NIV to stack (88% vs. 89%) (191). No difference was found between groups in the MIC achieved (bag = 1,344 ± 520 ml vs. NIV = 1,481 ± 477 ml, *p* = 0.33), or the PCF obtained when coughing from the inflated volume (bag = 186 ± 50 L/min vs. NIV = 199 ± 48 L/min, *p* = 0.33) (191). Similarly, the MICs obtained using IPPB or a MI-E device for inflation (190) or with stacked- and single-breath methods appear comparable (194).

In contrast, one case series of 282 consecutive patients attending a single centre reported that LVR using a bag and one-way valve resulted in a larger assisted inflation than LVR via volume-limited NIV or a bag without a valve, although both methods achieved assisted ICs above spontaneous VC [LIC = 2,069 ± 867 ml (range 320–5,400) vs. MIC = 1,712 ± 926 ml (range 30–5,100) vs. VC = 1,131 ± 744 ml (range 0–3,580)] (164). This retrospective data did not report the prevalence of bulbar dysfunction, and it is not known whether the order of assessment was randomised; if LVR has a short-term physiological effect on the respiratory function, this may influence subsequent values. Discord in the magnitude of volume augmentation with different LVR techniques between this large retrospective study and the smaller prospective studies reported in Table 3 may represent individual participant or clinician technique, highlighting the need for individualised assessment and titration.

Numerous studies have also evaluated the ability of LVR and other assisted inflation techniques to augment cough in people with NMD during periods of stability. Notwithstanding the previously detailed limitations of PCF as an outcome measure, the PCF when coughing from an inflated volume is larger than an unassisted volitional cough (Table 3). Furthermore, a positive association exists between the amount of volume recruited (MIC–VC difference) and the increase in augmented PCF (PCF_assisted_–PCF difference) (133, 170, 188, 194). One study has found this relationship extends to an optimal inflation capacity, with PCF_assisted_ declining past this point (82). The clinical significance of generating a larger PCF has not been definitively established.

## Immediate effect of LVR during periods of stability

Evaluation using opto-electronic plethysmography (OEP) demonstrates increased chest wall displacement attributable to expansion of the pulmonary-apposed (upper) and abdomen-apposed (lower) ribcage with LVR (192, 195). However, whilst LVR can augment volume (23, 29, 39, 40, 133, 164, 171, 173, 178, 191) and increase the PCF produced *during* the manoeuvre (23, 84, 85, 90, 93, 132, 133, 164, 170, 173, 176, 191, 196), these effects appear not to carry over to sustained consistent improvements in lung function following the LVR session. A number of studies have now investigated the immediate effects of a single session of assisted inflation therapy, using methods including LVR, IPBB, or mechanical insufflation (Table 4). The vast majority found no change in VC, lung compliance (*C*_L_), or *C*_rs_ after a therapy session (23, 29, 51, 177, 197–200), with a handful showing small improvements in some respiratory function measures that diminish within an hour (29, 30, 201).

**Table 4:** Studies investigating the immediate effect of a single session of assisted inflation in medically stable participants with NMD.

Author/s	Year	Number of participants	Population	Trial design	Hyperinflation therapy	Results
Sinha and Bergofsky (202)	1972	6	Restrictive CWD	Prospective pre-post intervention study	5 min IPPB	Dynamic *C*_L_ increased immediately post; sustained 1 h. Elastic WOB decreased
De Troyer and Deisser (197)	1981	10	Slow NMD	Prospective pre-post intervention study	15 min IPPB (also performed maximal inflations using VCV-NIV in sub-set)	No change in VC, FRC, *C*_L_
McCool et al. (51)	1986	14 NMD 6 Controls	Chronic SCI, slow NMD	Prospective pre-post intervention study	20 min IPPB	No change in *C*_rs_ (or *C*_L_ and *C*_CW_ in sub-set with measurements)
Simonds et al. (203)	1989	10	Restrictive CWD		5 min MI (volume or pressure NIV)	No change in accessible alveolar volume or oxygenation
Stiller et al. (204)	1992	5	Recent SCI		20 min IPPB (4 reps × 6 sets)	Statistically significant but small change in VC post therapy (0.4 ± 0.9 L)
Lechtzin et al. (198)	2006	14 MND 4 Controls	MND	Prospective pre-post intervention study	5 min MI (bilevel NIV)	No group change in *C*_L_
Laffont et al. (205)	2008	7	Recent SCI	Prospective pre-post intervention study	20 min IPPB	No change in dynamic *C*_L_ or WOB post
Guerin et al. (206)	2010	14	Slow NMD	Prospective pre-post intervention study	30 maximal inflations via IPPB	Improved tidal volume (*V*_T_) on EIT, up to 3 h post single session
Cleary et al. (177)	2013	26 PCF data 15 FVC data	MND	Prospective, pre-post cross-over study	5 maximal inflations via LVR	Improved PCF post, no change in FVC
Meric et al. (201)	2017	9	DMD	Prospective pre-post intervention study	15 maximal insufflations via MI-E	Statistically significant increase in VC post therapy, but small magnitude & diminished by 1 h. No change in *V*_T_
Molgat-Seon et al. (29)	2017	12 NMD 12 Controls	Slow NMD, Chronic SCI	Prospective pre-post intervention study	10 maximal inflations via LVR	Immediate increase in *C*_rs_, diminished by 1 h. No change in VC, FRC, PCF
Sarmento et al. (192)	2017	12 MND 12 Controls	MND, Controls	Prospective pre-post intervention study	3 maximal inflations via LVR	Increase in PCF, IC, and VC (measured via OEP) immediately post compared with baseline
Cesareo et al. (199)	2018	20	DMD	Prospective pre-post intervention study	5 × 5 maximal insufflations via MI-E (for lung volume recruitment)	No change in VC, *V*_T_, or PCF post MI-E. No change in lung volumes via OEP
Cleary et al. (207)	2021	29	MND	Prospective pre-post cross-over study	5 maximal inflations via LVR	LVR improved forced expiration, throat clearing, and other airway protection behaviours at 30 min
Pellegrino et al. (208)	2021	15	Slow NMD, MND	Prospective pre-post intervention study	3 maximal inflations via LVR	Small improvement in dypnoea (Borg score) up to 2 h post. No change in SpO_2_ or ventilation distribution (FOT)
Veldhoen et al. (30)	2022	67	Paediatric and adult NMD	Prospective pre-post intervention study	∼5 reps × 3 sets maximal inflations via LVR (*n* = 48), or ∼5 reps × 5 sets via MI-E (*n* = 19)	LVR: Immediate increase in VC, diminished by 1 h. MI-E: Immediate increase in VC, diminished by 2 h. No change in PEF with either
Casaulta et al. (200)	2022	8	Paed NMD	Prospective pre-post intervention study	∼5 reps × 5 sets via MI-E	No change in VC, PEF, LCI, or global inhomogeneity index (EIT)
Sheers et al. (23)	2022	80	Slow NMD MND	Prospective pre-post intervention study	5 reps × 2 sets maximal inflations via LVR	Increase in *C*_rs,_ LIC, and PCF_LIC_. No change in VC, FRC, or static lung volumes, PCF

The shaded rows highlight studies employing an LVR kit.

CWD, chest wall disease; NMD, neuromuscular disease; SCI, spinal cord injury; MND, motor neurone disease; DMD, Duchenne muscular dystrophy; IPPB, inspiratory positive pressure breathing; VCV-NIV, volume-limited mode (i.e., volume-controlled ventilation) of non-invasive ventilation; MI, mechanical insufflation; NIV, non-invasive ventilation; LVR, lung volume recruitment; MI-E, mechanical insufflation-exsufflation; *C*_L_, lung compliance; WOB, work of breathing; VC, vital capacity; FRC, functional residual capacity; *C*_rs_, respiratory system compliance; *C*_CW_, chest wall compliance; *V*_T_ = tidal volume; EIT, electrical impedence tomography; FVC, forced vital capacity; PCF, peak cough flow; OEP, opto-electronic plethysmography; SpO2, oxygen saturation; FOT, forced oscillation technique; PEF, peak expiratory flow; LCI, lung clearance index; EIT, electrical impedence tomography; PCF_LIC_, PCF from LIC.

With regard to studies that have evaluated the LVR technique, Cleary and colleagues conducted a cross-over trial, comparing a single session of LVR (5 maximal inflations and 2 assisted cough manoeuvres) with a control period in 29 participants with ALS/MND who used LVR at home. The forced vital capacity, PCF, and sniff nasal inspiratory pressure (SNIP) were measured at baseline, 15 and 30 min post therapy. A significant interaction effect was found; however, this represented a statistically significant difference in FVC between groups at 15 min and not a difference in the change over time. Unassisted PCF increased post-LVR at 15 min and 30 min after the single session compared with baseline, with significant between-group differences. The authors postulated that the mean increase of 54 L/min may represent an improvement in compliance (177); however, given the lack of concomitant increase in lung volume and that *C*_rs_ was not assessed, this change may also have reflected PCF measurement variability. The same authors recently showed a benefit of LVR on the expiratory flow rates measured using a peak flow metre during volitional airway clearance and airway protection behaviours in the same patient cohort (207). Whether assisted inflation therapy improves airway protection and swallow function on videofluoroscopy remains to be seen.

A pre-post intervention study was conducted in 12 participants with slowly progressive NMDs and 12 healthy controls (29). Lung volume recruitment comprised 10 maximal inflations, resulting in a significant increase in volume (MIC) and PEF_LVR_ in the participants with NMD during the manoeuvre. Between-group differences were observed at all time points, with PCF and all lung volumes except that RV was significantly lower in the NMD cohort. However, in contrast to the Cleary study in people with ALS/MND (177), neither group demonstrated any effect of LVR on unassisted PCF or lung volumes over time (29). Respiratory system compliance (*C*_rs_), measured using the pulse inflation method (209), was lower in the NMD group relative to controls. Immediately following a single session of LVR, there was an improvement in *C*_rs_ (37 ± 5 to 50 ± 7 ml/cmH_2_O) representing a change of 40 ± 10%; however, the levels returned to baseline within an hour. The individual data suggest that there may be responders and non-responders; 8 out of 12 participants obtained an improvement greater than 20% of baseline, whereas the other third had smaller or a negative response. Another interpretation may be that the findings represent test/re-test variability. The authors concluded that resolution of atelectasis was not the mechanism by which *C*_rs_ increased, as the static lung volume did not change (29).

Two large prospective pre-post intervention studies have since been conducted, with both confirming no change in unassisted PCF or expiratory flow following a session of assisted inflation therapy in people with NMD. Veldhoen and colleagues took children and adults who performed regular airway clearance using either LVR or MI-E and measured FVC and PEF before, after, 1 h, and 2 h following therapy. A small improvement in FVC was observed immediately after LVR [mean (95% CI) increase of 90 (45–135 ml)], with values returning to baseline within an hour. A similar increase in FVC was shown with MI-E [mean = 59 (10–109 ml)], representing a <10% change from mean baseline values in the SMA subgroup, which persisted at 1 h but had diminished within 2 h (30). In the largest study to date, Sheers et al. found no long-standing change in VC or static lung volumes following LVR. Lung inflation capacity and PCF_LIC_ did improve immediately following therapy compared with baseline, which may reflect a practice effect in their naïve cohort (23). This study comprehensively measured the respiratory function as per Molgat-Seon et al. (29), and similarly reported an improvement in *C*_rs_, albeit smaller in magnitude (23).

Research on other alternative methods of assisted inflation are summarised in Table 4. Whilst some studies suggest an improvement in *C*_L_ and/or lung volume following a single session of therapy, others found no change in these parameters. Given the small number of the participants involved (ranging from 6 to 20), this lack of consensus is not surprising; it is plausible that there are responders and non-responders, perhaps related to individual baseline characteristics such as degree of lung volume restriction or disease duration. Alternatively, this variation may be random and reflect no true effect. The studies by De Troyer (197), Molgat-Seon et al. (29), Veldhoen (30), and Sheers (23) provide the most comprehensive evaluation of assisted inflation therapy on respiratory mechanics, and three found no demonstrable benefit on VC or unassisted PCF. The growing body of literature suggests that whilst small or short-lasting increases in *C*_rs_ may occur with recruitment techniques in medically stable people with NMD, there is minimal evidence that this improves lung volumes or ventilation. The effect of LVR or hyperinflation in people with NMD during episodes of RTI is a different proposition to during stable periods. The few studies that have been conducted suggest clinical utility, improved secretion clearance, and PCF; however, the impact on lung function is unknown (135, 210, 211).

## Regular LVR therapy

Research evaluating the regular use of assisted inflation therapy has, until recently, been largely retrospective or uncontrolled in design (Table 5), but nonetheless has contributed to the recommendations that daily LVR therapy be performed routinely by people with NMD (17, 19–22). The latter study designs are limited by the absence of a control or comparator cohort to account for the natural history of respiratory function decline in progressive NMDs, and the use of self-report to determine the performance of daily therapy, which in other areas has been shown to be unreliable (212, 213). Furthermore in the cohort studies, only those patients who self-selected to return to clinic are represented, and other potential confounders such as timing of NIV, advances in ventilation strategies, pharmacological management, and evolution of a multidisciplinary team approach are present.

**Table 5:** Studies investigating the effects of regular assisted inflation in participants with NMD.

Author/s	Year	Number of participants^a^	Population	Trial design	Therapy and follow-up period	Results
Houser and Johnson (214)	1971	14	DMD, paed	Randomised matched-pair study	6 min IPPB 5 days/week (*n* = 7) vs. control (*n* = 7), for 3 months	No difference in rate of FVC decline
Adams and Chandler (215)	1974	3	DMD, paed	Case series	Swimming + IPPB programme for 11 months	VC improved during the programme, decreased during vacation periods
Huldtgren et al. (216)	1980	12	Sub-acute cervical SCI	Case series	10 reps LVR inflations + EMST 10 reps EMST 10 reps IMST Training 5x/week for ∼6 weeks	Improved VC, MIP, and MEP after respiratory rehabilitation training programme
Simonds et al. (203)	1989	10	Restrictive CWD	**Prospective uncontrolled trial**	5 min MI (6 participants = volume, 4 = pressure NIV), ×2–3/day for 9 months.	No change in VC or TLC overall. Small, statistically significant increase in VC in volume group
Kang and Bach (163)	2000	43	MND, slow NMDs	Retrospective case series (date range not stated)	108 cases (65 single Ax, 43 multiple visits) Prescribed LVR 10–15 inflations 3x/day if VC <2 L Follow-up period not stated	All reported using at least 2×/day: 30/43 increased MIC over time ◊ VC unchanged, assisted PCF increased 13/43 decreased MIC over time ◊ VC and assisted PCF fell
Miske et al. (217)	2004	62	Slow NMD	Retrospective case series (1998–2001)	MI-E prescribed for home use as required (3–5 reps × 3–5 sets)	Descriptive study: MI-E well tolerated with minimal side effects in 90% of cohort
Bach et al. (174)	2007	47	DMD	Retrospective case series (1996—end date not stated)	78 cases (31 single Ax, 47 multiple visits) Prescribed LVR 10–15 inflations 3x/day Follow-up period 7–169 months	31/47 reported using at least 2×/day: MIC increased, VC fell over time in 31 patients; no comparison with 16 patients who did not perform routinely.
Bach et al. (164)	2008	46	MND, slow NMDs	Retrospective case series (2005—end date not stated)	282 cases (204 single Ax, 78 multiple visits) Prescribed LVR 10–15 inflations 3x/day Follow-up period not stated	46/78 had follow-up data: MIC and LIC increased, VC fell over time.
Laffont et al. (205)	2008	14	Recent SCI	Randomised cross-over trial	20 min IPPB 2×/day, 5 days/week for 2 months vs. control	No difference in VC, lung volume, dynamic *C*_L_ between IPPB or no IPPB periods
Nygren-Bonnier et al. (218)	2009	11	SMA (6–16 years)	**Prospective uncontrolled trial**	GPB training 4×/week for 8 weeks 10 maximal inflations/session	5/11 participants able to learn GPB. Improved chest expansion, PEF, and inspiratory VC in *n* = 4 completed participants
Nygren-Bonnier et al. (219)	2009	25	Chronic cervical SCI	**Prospective uncontrolled trial**	GPB training 4×/week for 8 weeks 10 maximal inflations/session	20/25 participants able to learn GPB. Improved chest expansion, VC, and static lung volumes post 8 weeks
Johansson et al. (220)	2011	7	Chronic cervical SCI	**Prospective uncontrolled trial**	GPB training 4×/week for 8 weeks 10 maximal inflations/session	No difference in VC pre and post 8 weeks. Some positive effects on speech
McKim et al. (40)	2012	22	DMD	Retrospective cohort study	Prescribed 2×/day LVR: 3–5 maximal inflations/session Compared RFT data pre-LVR (median 34 months) with post-LVR (45 months)	22 reported adherent with 2×/day LVR Rate of FVC decline slowed post-LVR: pre-LVR 4.7 vs. post-LVR 0.5%pred/year
Srour et al. (175)	2013	35	Multiple sclerosis	Retrospective case series (1999–2010)	79 cases (44 single Ax, 35 multiple visits) Prescribed 2×/day LVR: 5 maximal inflations/session if FVC <80% and trial of LVR improved RFT (MIC > VC) Median follow-up 13 months	Of 35 patients prescribed regular LVR and multiple data: Rate of FVC decline slower in group who achieved PCF_LVR_ > PCF at baseline
Moran et al. (221)	2014	10	Paed NMD	Retrospective cohort study	Home MI-E as prescribed by allied health professional	Fewer days hospitalised post MI-E, positive qualitative feedback
Phillips et al. (222)	2014	6	Paed NMD	Prospective cohort study	Home MI-E as prescribed by allied health professional	Fewer days hospitalised post MI-E, positive qualitative feedback
Marques et al. (171)	2014	22	Slow NMDs, paed	**Prospective uncontrolled trial**	4–6 months of 3×/day LVR 3–4 maximal inflations/session	18/22 completed No change in FVC or MIC. Unassisted and assisted PCF increased
Kaminska et al. (178)	2015	24	MND, slow NMDs	**Prospective uncontrolled trial**	3 months of 2–4×/day LVR 3–5 maximal inflations/session	19/24 completed ◊ 14 willing to continue LVR post study period FVC fell, LIC and LIC—FVC increased over time No change in PCF or QoL
Rafiq et al. (88)	2015	40	MND	**Randomised controlled trial**	1 year of LVR *or* MI-E 3–5 maximal inflations 2×/day	Primary outcome = RTI. No difference b/w groups in RTI rate No difference in survival, QoL Adherence: 71% LVR, 53% MI-E
Jeong and Yoo (223)	2015	14 LVR 12 IS control	Recent SCI	**Randomised controlled trial**	5 days/week for 6 weeks of LVR *or* IS 20 repetitions 2×/day	FVC and PCF increased over time in both groups, but PCF improvement greater with LVR > IS
Stehling et al. (224)	2015	21	Slow NMD, paed	Retrospective cohort study (2009–2012)	3 maximal inflations via MI-E repeated in sets for 10 min, 2×/day. Analysed VC for 2 years pre and 2 years post initiating MI-E at home	VC increased within the first year post MI-E initiation (mean relative improvement = 28%)
Moran et al. (225)	2015	7	Paed DMD or SMA	Qualitative research	Home MI-E as prescribed by allied health professional	Positive and negative impacts of home MI-E on lifestyle identified
Mahede et al. (226)	2015	37	Slow NMD	Cohort study (2007–2011)	Home MI-E as prescribed by allied health professional. Mean duration 2.3 years	MI-E at home improved self-reported health and reduced ED presentations (qualitative data, health record linkage)
Katz et al. (39)	2016	16	DMD	Retrospective cohort study (1991–2008)	Prescribed 2×/day LVR: 3–5 maximal inflations/session Median follow-up = 6.1 years	LIC-VC increased 0.02 L/year LIC increased and FVC stable/rate of FVC decline slowed post-LVR: pre-LVR 4.5 vs. post-LVR 0.5%pred/year
Chiou et al. (227)	2017	151	DMD	Retrospective case series (1996–2015)	232 cases (81 single Ax, 151 multiple visits) Prescribed LVR 10–15 inflations 3x/day once VC plateaued (53 cases)	151 patients: rate of VC decline = 8.8% of plateau VC/year (includes 53 below) 53 patients prescribed LVR: rate of VC decline = 8.5% of plateau VC/year
An and Shin (228)	2018	24	SCI (mean onset ∼1 month post injury)	**Randomised controlled trial**	3 days/week for 4 weeks of LVR + IMST *or* IS + IMST LVR or IS: 15 reps × 3 sets, plus IMST = 15 min	Improvement in FVC and MIP with both groups, but greater with LVR + IMST > IS + IMST. PCF improved over time, with no difference between groups
Chatwin and Simonds (181)	2020	181	Slow NMD	Retrospective case series (2014–2018)	181 patients with MI-E at home and prescribed daily use (includes service provision, MI-E criteria, use, settings)	Yearly adherence data on 137: median days used = 60%, 1.8 sessions/day, 2.3 min/session.
Veldhoen et al. (229)	2020	37	Paed NMD (eg. SMA)	Retrospective case series	MI-E commenced for daily use as per local protocol. Recommended dosage: 5 resp × 3 sets 2×/day	Fewer RTI related admissions in period post initiation of MI-E.
Sawnani et al. (230)	2020	31	Congenital MDs (5–21 years)	**Randomised controlled trial**	1 year of Hyperinflation via MI-E device *or* control MI-E: 15 min 2×/day Control: Routine care Follow-up at 4, 8, and 12 months	No difference in primary outcome (change in FVC) between groups at endpoint (1 year). No difference in QoL. Overall adherence 44%
Katz et al. (28)	2022	66	DMD (6–16 years)	**Multicentre randomised controlled trial**	2 years of conventional treatment + LVR *or* conventional treatment alone LVR: 3–5 maximal inflations 2×/day Follow-up 6, 12, 18, and 24 months	No difference in primary outcome (change in FVC %pred) between groups at endpoint (2 years). Adherence 41%
Sheers et al.^b^ (231, 232)		73	Slow NMD MND	**Randomised controlled trial**	3 months of LVR *or* control LVR: 5 reps × 5 sets maximal inflations 2×/day Control breathing exercises: 5 reps × 5 sets 2×/day Follow-up 1, 2, and 3 months	Improvement in primary outcome (change in LIC) between groups at endpoint (3 months). No treatment effect on lung volumes, *C*_rs_, or QoL. LVR Adherence 45%

DMD, Duchenne muscular dystrophy; paed, paediatric cohort; CWD, chest wall disease; MND, motor neurone disease; NMD, neuromuscular disease; SCI, spinal cord injury; MDs, muscular dystrophies; IPPB, inspiratory positive pressure breathing; MI, mechanical insufflation; NIV, non-invasive ventilation; LVR, lung volume recruitment; MI-E, mechanical insufflation-exsufflation; IS, incentive spirometry; IMST, inspiratory muscle strength training; EMST, expiratory muscle strength training; Ax, assessment; RFT, respiratory function test; VC, vital capacity; FVC, forced vital capacity; TLC, total lung capacity; MIC, maximum insufflation; *C*_rs_, respiratory system compliance; QoL, quality of life; PCF, peak cough flow; ED, emergency department.

The shaded rows highlight the studies employing an LVR kit. Trial design notes in **bold** signify prospective studies.

^a^ Number of participants with longitudinal data.

^b^ Abstract of conference proceedings; manuscript currently under review.

### Retrospective adult cohort data

The earliest, retrospective works represent the clinical practice of two groups: Bach and colleagues and McKim and colleagues. The four case series published by Bach et al. describe their cohorts of patients with DMD (174, 227) or heterogeneous NMD (including DMD and ALS/MND) (163, 164). Included are all patients who underwent initial evaluation, which included testing of VC, MIC and/or LIC, PCF, and assisted PCF. If VC was less than 70%–80% predicted or 2 L, the patients were prescribed LVR three times a day. Those who returned for re-evaluation were monitored with repeat respiratory function testing. If not already performing LVR, it was initiated once the VC criterion was met, or in the case of DMD, once VC plateaued. The published data were obtained from retrospective chart reviews although it is not clear if these papers represent the same data or different patients.

In the patients who returned for routine evaluation, it was observed that MIC could improve over time, even in the face of declining VC (164, 174). In one study, VC remained unchanged in those patients who increased MIC over the follow-up period, whereas it fell in the patients who also demonstrated a declining MIC. A small but statistically significant improvement in assisted PCF was also reported (222 ± 84 to 258 ± 96 L/min, difference = 42 ± 66 L/min, *p *< 0.01) (163). The interpretation of the authors was that regular LVR can improve the respiratory function in the context of progressive NMD, and concluded that this therapy “is indicated for all NMD patients with diminishing VC” (164).

However, these retrospective cohort studies have a number of additional limitations that cast doubt on the strength of the conclusions made, including poorly defined follow-up periods and incomplete data sets with the potential for retention bias. Furthermore, categorising patients and comparing those who increased vs. decreased MIC over time without considering the underlying disease, in a sample of 43 participants with a mixture of rapidly and slowly progressive NMDs (163), introduces a considerable confounder. These papers, representing small and select patient populations, raised the notion that MIC may improve over time in some people with NMD. The authors proposed that it signified greater lung and chest wall “range of motion” and resulted in improved cough effectiveness that can decrease the risk of pneumonia (163). The increase in MIC observed may however reflect a practice effect, rather than a change in respiratory system mechanics. The significant retention bias also limits the interpretation of the results; most patients elected not to return to the clinic (35%–72% of the published cohorts had a single assessment only); therefore, the change in the respiratory function is not available for comparison, and may be similar to those who stated that they routinely performed LVR.

The work by McKim, Katz, and colleagues in people with DMD also suggested a possible long-term benefit of regular LVR (39, 40). Their initial paper of 22 patients who had been initiated on twice-daily LVR demonstrated a slower rate of FVC decline in the 45 months after therapy commenced compared with the 34 months prior (pre-LVR = 4.7% predicted per year vs. post-LVR = 0.5% predicted per year). The authors acknowledged the limitations of self-reported LVR use and the potential for NIV use to confound lung function changes, and called for a prospective RCT to support their observational effects and aid translation (40). The follow-up study of 16 participants from the same cohort for a median of 6 years suggested that the improvement in MIC–VC difference was maintained for up to 10 years after LVR initiation, with concomitant stability in PCF. Maximal insufflation capacity also increased slightly during the first 4–5 years post initiation of LVR. Whilst the authors hypothesised that LVR may preserve *C*_rs_, they did not measure this (39). Nonetheless, the observation that FVC did not significantly fall over time in the setting of worsening MEP values (40) would support the hypothesis that regular LVR may maintain lung and/or chest wall “flexibility”.

The same group also examined the effect of LVR in people with multiple sclerosis and found that of the 35 people who returned to the clinic, the rate of decline of FVC was slower in those who could perform the technique and were prescribed daily LVR, compared with those who were not. Although FVC and PCF values declined, MIC remained stable but did not increase. The participants prescribed routine therapy had lower baseline FVC and unassisted PCF, suggesting that LVR may be more beneficial in people with more restricted pulmonary function (175). Alternatively, given that the participants in the LVR group were more severe than those who did not perform LVR routinely, the decline in the latter group may represent natural disease progression, again highlighting the need for prospective controlled data.

### Retrospective paediatric cohort data

There are no data in infants or young children on the effect of longer-term LVR using a manual resuscitation bag, but several retrospective studies have focussed on determining the impacts of long-term use of MI-E on the quality of life (QoL) or qualitative outcomes (222, 225, 226) and quantitative outcomes such as hospitalisations (221, 222, 226), or spirometry (222). Mechanical insufflation-exsufflation is a combinatorial therapy; the insufflation element (the “I”) is inflation therapy; however, coupled with rapid exhalation (the “E”), the therapeutic aim frequently includes secretion clearance. In the paediatric literature, it appears that both aims are targeted clinically; regular MI-E when stable aimed primarily at maintaining lung volume in the face of progressive weakness and growth, and increasing MI-E frequency when unwell to assist with airway clearance and expectoration.

Overall retrospective MI-E study findings in paediatrics are relatively consistent with a reduction in hospital admissions, days hospitalised or emergency department presentations. All studies are limited by their retrospective nature, limited reporting of MI-E prescription, and, if usage frequency was available, it was patient/caregiver self-report. Despite this, the studies suggest overall that MI-E may impact hospitalisation, either through reduced infections or through greater ability to manage episodes at home.

The quality of life data are also reasonably consistent, with the participants in all studies identifying a number of health and lifestyle benefits that led to widespread acceptance of the device by both families and children with NMD. Though multiple positive impacts are identified in these studies, MI-E use was not without its challenges. These included portability, power dependence, and increased pressure on those caregivers trained in MI-E administration amongst the most common.

Moran et al. explored these findings further using semi-structured interviews to investigate the experiences of a small cohort (11 participants) of both parents and children regularly using MI-E at home (225). They identified both positive and negative impacts of MI-E use, highlighting the need for wider consideration and discussion of the devices impact on the family life prior to its initiation.

The role of MI-E to both augment volume and assist with secretion management makes it impossible to partition out the relative benefits of regular lung volume recruitment vs. any benefit of a change in respiratory tract infections and/or hospitalisation. This limitation is particularly apparent in the paediatric literature.

### Prospective studies of LVR usage

Two prospective, uncontrolled studies examined the respiratory function 3–6 months after prescribing LVR in NMD populations naïve to therapy (171, 178). The dosage was similar in both studies, and consistent with that used by the McKim and Katz group (39, 40). Marques and colleagues studied younger participants with NMD who were not on NIV (mean age of 15 years, range 7–23 years) and found no change over time in FVC or MIC, but an improvement in PCF measures. The authors reported a significant improvement in FVC when nine patients without scoliosis were analysed separately; however, the mean magnitude of this increase was 91 ml (or <5% of baseline) (171) and unlikely to be clinically significant. Adherence was not assessed.

Twenty-four older participants (mean age of 54 years) with a diagnosis of ALS/MND, post-polio syndrome, or myotonic dystrophy were investigated by Kaminska et al. Adherence diaries, questionnaires assessing QoL, and acceptance of LVR were measured 3 months after initiating twice-daily LVR (178). There was a small but statistically significant decline in FVC of 82 ml (95% CI = −159, −5 ml) in the 19 patients who completed the study, with a trend to increasing LIC [154 ml (−13, 322 ml)]. Consequently, the LIC–FVC difference increased over time [243 ml (65, 420 ml)], with a greater magnitude of effect in the 12 participants who reported performing the therapy twice a day [430 ml (126, 558 ml)]. No changes were detected in QoL over the 3-month period (178). The authors interpreted this finding to mean that the twice-daily exercises did not place additional burden on people's care; however, it may also indicate that the participants perceived no symptomatic improvement in their health-related QoL. Given that only half of the recruited cohort performed LVR as prescribed and 10 out of the 24 recruited participants were not willing to continue after the study concluded (178), the latter interpretation may be more possible. The qualitative elements of this feasibility study raise important questions regarding the perceived benefit vs. the burden of performing therapy trade-off that all individuals consider when undertaking routine exercise.

These themes were also evident in a randomised comparison study comparing a year-long trial of LVR with MI-E in participants with ALS/MND. Of the 21 participants in the LVR arm, 71% reported performing two sessions a day compared with 53% of the MI-E group. Of the 15 participants who did not use a technique as prescribed, 11 had severe bulbar impairment. No change was noted in the carer strain or QoL indices. There was a low event rate of RTI in both groups (the primary outcome), and respiratory function analysis was limited to VC and PCF. No differences were noted between groups in the average rate of decline per month. Two major limitations of this study were the commencement of assisted inflation therapy at the same time as NIV initiation, a substantial confounder given the impact of NIV on the respiratory function, symptoms, and QoL in people with ALS/MND; and the lack of a control or active control group (88).

A comparator intervention was also included in the two RCTs that assessed the effect of LVR in people with cervical SCI. The participants were allocated to LVR or incentive spirometry (with the addition of inspiratory muscle strength training in one study) (228) for between 4 and 6 weeks (223, 228). In both trials, FVC and PCF increased over time; Jeong et al. reported no between-group difference in FVC but a larger PCF improvement in the LVR group (baseline = 204 ± 129 L/min to post = 261 ± 126 L/min) compared with the incentive spirometry arm (240 ± 142 L/min to post = 249 ± 110 L/min) (223). In contrast, An and Shin found a greater FVC improvement in the LVR group compared with incentive spirometry, but no difference in PCF response (228). The applicability of these findings to medically stable NMDs is unclear given the time course post injury and natural history of recovery in SCI.

Recently, Katz and colleagues published the results of their RCT in children with DMD, a multicentre, single-blinded trial of standard care or standard care plus twice-daily LVR over 2 years. A total of 66 boys with median age of 11.5 (interquartile range 9.5–13.5) years and with normal to very mild respiratory impairment [median FVC 85% (73%–96%)] completed the study. No difference in the change in % predicted FVC was seen between the two arms, or in any of the secondary outcome measures that included the MIC–VC difference, MIP, MEP, or TLC. Adherence to therapy was sub-optimal (41% of participants); however, after adjusting for usage, the results were similar (28).

The prospective studies detailed above are consistent in their findings, with all reporting no demonstrable effect of regular LVR on VC, MIC or LIC, or QoL in the populations studied. Interestingly, Sawnani and colleagues used a MI-E device to deliver regular assisted inflation therapy to 34 children with muscular dystrophy and worse respiratory function (56% predicted FVC) in their randomised controlled trial (RCT) and did find an improvement in FVC at interim time points, but not at the 1-year mark (230). It is plausible that regular LVR may be beneficial for certain groups of people with NMD, i.e., if a certain level of respiratory impairment has been reached, or that benefits on respiratory function decline may take years to become apparent. However, current best-available evidence would suggest that daily volume recruitment has no effect if commenced too early, that is, before an as yet unknown threshold.

## Current research gaps and potential developments

The current literature demonstrates that LVR clearly has basis in physiology; however, there is not enough data to demonstrate its effectiveness as a regular maintenance therapy in most people living with NMD. Cohort data are biased, but show that in “adherers”, those who remain within the cohort for analysis, LIC seems to be maintained over time despite ongoing reduction in VC (39, 40, 163, 164, 174). It has been proposed by Bach, Kang, and others that LIC reflects “range of motion”, and that assisting inflation to the maximum, tolerable insufflation capacity of the participant is analogous to “passive range” exercises (39, 133, 163, 170, 227). If passive stretch using LVR exercises maintains LIC over time whilst VC declines with progressive weakness, then it would be expected that total respiratory system compliance (i.e., *C*_rs_) would be maintained over time. A study currently underway from our group is comparing 3 months of regular LVR with an active control (https://www.anzctr.org.au, ACTRN12615000565549). Full clinical trial results will be available in late 2023, but preliminary results from Sheers et al. in 76 participants with heterogeneous NMDs (ALS/MND = 34%) and a mean baseline VC 41% predicted demonstrated a significant improvement in LIC of 12% (95% CI = 4, 21%) in the LVR group at 3 months compared with control [mean change 0% (−6, 7%)], but no between-group difference in *C*_rs_ (231, 232). Further controlled trials and comprehensive longitudinal cohorts are required to resolve whether any observed changes in LIC represent “true change” or are attributable to learning or other effects. Similarly, further intra- and extra-pulmonary system compliance data collected over a longer time period are required to understand change over time with regular LVR therapy, if indeed a long-term benefit exists.

Most of the LVR literature has used uncomplicated, predominately spirometric measures of lung function as outcome measures. Whilst this has meant that the experiments could easily be undertaken in the clinic or community, it has inevitably also meant that we have little information about mechanisms of effect (or not). Mechanisms of action have generally been theorised from “first principles” in the literature, whereas more sophisticated measures would provide better empirical evidence of efficacy. For example, the forced oscillation technique (FOT) measures impedence of the respiratory system (*Z*_rs_), including resistance (*R*_rs_) and reactance (*X*_rs_) components, and can detect ventilation inhomogeneity and alveoli closure (233). At low FOT frequencies, *X*_rs_ is largely influenced by the elastic recoil of parenchyma and respiratory tissues, and is well correlated with the proportion of ventilated lung seen on computed tomography scans (234). Gauld and colleagues demonstrated that FOT is feasible in young children with SMA (235), and further work to determine the utility of this non-invasive, non-volitional measure in the NMD population is warranted.

Similarly, there has been recent interest in electrical impedence tomography (EIT) to assess short-term changes with assisted inflation therapies (200, 206). This technique images the impedence changes between electrodes placed around the thorax, and can measure ventilation distribution (236). The lung clearance index (LCI), calculated from the multiple-breath washout technique, is another index of ventilation inhomogeneity (237). If therapies improve ventilation by re-expanding decruited lung or clearing pulmonary secretions, then a signal may be apparent on pulmonary function tests such as FOT, EIT, LCI, and/or labelled ventilation-perfusion imaging. Opto-electronic plethysmography (OEP) assesses ribcage kinematics and externally derived lung volume (238), and has already been used by some authors to measure chest wall expansion and the relative contribution of upper and lower components of the ribcage (192, 199, 201). Combining measures of ventilation with this assessment of the chest wall may help to unpack the internal and external mechanisms that LVR is hypothesised to change.

As with much physical therapy research, accurate and objective recording of LVR “dose” of therapy is challenging. Adherence with LVR is variable across all of the reported, longer-term research, and with the exception of Chatwin et al. (181), Sawnani et al. (230), and Katz et al. (28), is based on subjective self-report (Table 5). A recent paper by Naughton et al. described an LVR counter that can be attached to the commercially available LVR kit (239), and this device was used to measure adherence in the Sheers et al. experiment above (231, 232). Briefly, the device consists of a battery-powered data recorder that logs pressure activation at two points in the LVR bagging circuit. In this way, the device logs “time at pressure”, analogous to usage with home continuous positive airway pressure devices and gives a true estimate of lung insufflations. This device was developed in-house, and we are unaware of a similar, commercially available adherence monitor. A commercially available LVR counter would appear critically important to support the experiments that test real-world objective LVR adherence, estimates of dose-response, acceptability, and other questions.

Given the current advances in the pharmacological management of conditions such as SMA (and potentially other neurodegenerative diseases such as ALS/MND in the coming years), the concomitant change in clinical presentation, shift in care objectives, and growth in international recommendations for LVR use, it is doubtful that an RCT design of adequate duration can be conducted to answer the questions raised above. Prospective longitudinal studies with *a priori* hypotheses in larger numbers of people living with NMD are required, and should include clinical outcomes such as hospitalisation, time to ventilatory support, and mortality. Models for this approach already exist, for example, the 20-year cohort study by Berlowitz and colleagues examining the effect of NIV on survival in MND (7), and the work by Rose et al. quantifying healthcare utilisation in a Canadian adult NMD cohort (67). Reaching consensus on respiratory outcomes, collecting respiratory function regularly as part of standardised clinical care, recording this in a national NMD registry, utilising hospital presentation, mortality, and other “big data”, and collaborating widely are imperative, with quantitative and qualitative comparative clinical trials complementing this programme of research. A critically important element of any such cohort would be the inclusion of measures of therapy burden, LVR usage, patient reported outcome (PROM), and experience (PREM) measures. A consideration of health economic modelling would further strengthen the impact of any cohort finding.
